# Smart Furniture-Embedded Bed-Interaction and Multimodal Room Sensing with Sleep-Aware Hierarchical Fusion for Control-Oriented Bedroom State Recognition

**DOI:** 10.3390/s26185743

**Published:** 2026-09-10

**Authors:** Xinying Ru, Yumei Bai, Maoxu Wang, Hui Wan

**Affiliations:** 1 College of Home Furnishing and Art Design, Northeast Forestry University, Harbin 150040, China; 2 College of Computer and Control Engineering, Northeast Forestry University, Harbin 150040, China

**Keywords:** furniture-embedded sensing, multimodal sensor fusion, sleep-aware state recognition, smart bedroom, indoor environmental quality, bed-interaction sensing, edge inference, bedroom environmental control

## Abstract

**Highlights:**

**What are the main findings?**

- A bed-centered multimodal sensing framework was developed by integrating furniture-embedded bed-interaction sensing with bedside IEQ, room-context, and time features to recognize eight control-oriented sleep-related bedroom states.
- The proposed SA-HMF model achieved strong subject-independent performance, with 0.925 Accuracy, 0.874 Macro-F1, and reliable extraction of in-bed, final out-of-bed, nocturnal out-of-bed, and wake-up transition events.

**What are the implications of the main findings?**

- Furniture-embedded bed-interaction sensing provides essential in-bed and movement information, while room-context and time cues remain necessary for distinguishing unoccupied and occupied-out-of-bed states.
- The edge-deployable SA-HMF model supports privacy-preserving local inference and exploratory downstream smart bedroom applications.

**Abstract:**

Reliable control-oriented bedroom state recognition is needed because room-level occupancy sensing cannot distinguish in-bed, out-of-bed, movement, nocturnal bed-exit, and wake-related conditions. This study developed a privacy-preserving bed-centered sensing framework and a sleep-aware hierarchical multimodal fusion model, termed SA-HMF, for recognizing eight operational bedroom states (S0–S7). SA-HMF uses a bed-conditioned, quality-aware asymmetric architecture: bed-load and bed-mounted accelerometry are first encoded into a bed-interaction representation, which then conditions the fusion of bedside indoor environmental quality, room-context, temporal, and sensing quality information. Data were collected from 20 healthy adults across 127 valid participant-nights in one experimental bedroom. The recordings generated 342,684 overlapping 30 s windows using a 10 s stride; these windows were treated as correlated model instances rather than independent observations. Under the primary subject-independent split, SA-HMF achieved 0.925 Accuracy, 0.874 Macro-F1, 0.927 Weighted-F1, and 0.864 Balanced Accuracy. LOSO evaluation yielded a Macro-F1 of 0.852 ± 0.043, with a participant-level 95% confidence interval of 0.833–0.870. Using non-overlapping 30 s windows, SA-HMF retained 0.918 Accuracy, 0.865 Macro-F1, and 0.856 Balanced Accuracy. Retrospective event extraction F1-scores ranged from 0.846 to 0.950. The optimized model was 2.7 MB and required 15.6 ± 3.2 ms per inference. These results support feasibility and transfer to unseen healthy participants within the tested bedroom and sensing configuration. External validation across bedrooms, beds, sensing installations, and populations remains necessary.

## 1. Introduction

Reliable nocturnal state sensing is a prerequisite for sleep-aware bedroom environmental control. With the development of smart homes, indoor environmental quality monitoring, and edge intelligence, the bedroom is gradually evolving into an intelligent microenvironment capable of environmental sensing, state recognition, and device coordination. In nocturnal bedrooms, IEQ variables such as air temperature, relative humidity, CO_2_ concentration, illuminance, and ventilation status can affect sleep environment comfort, whereas lighting, air-conditioning, and ventilation actions may also disturb sleep [1,2,3,4,5]. Therefore, smart bedroom control requires not only room occupancy detection but also finer-grained recognition of sleep-related bedroom states, such as in-bed, occupied-out-of-bed, stable sleep-related state, movement/turning, nocturnal out-of-bed, and wake-up transition.

Existing smart home systems commonly rely on room-level sensors, such as PIR, door-contact, temperature and humidity, CO_2_, and illuminance sensors, for occupancy detection and environmental monitoring [6,7,8]. These sensors are easy to deploy and can reflect room occupancy, entry/exit events, and environmental changes. However, they cannot directly capture human–bed interactions. Consequently, room-level sensing alone often fails to distinguish “present in the room but not in bed”, “awake in bed”, “stable sleep-related state”, “brief movement”, “nocturnal out-of-bed” event, and “wake-up transition”. Bed-interaction sensing can complement this limitation by capturing in-bed status, bed-exit events, bed-load stability, and body movement, while bedside IEQ and room-context sensing provide environmental and activity cues needed for control-oriented interpretation.

### Related Work and Research Positioning

Existing sleep monitoring studies have investigated wearable, bed-based, mattress-integrated, and contactless sensing systems for sleep assessment, sleep stage estimation, respiration monitoring, heart rate estimation, posture analysis, and sleep quality evaluation [9,10,11,12,13,14,15,16,17,18,19,20]. Wearable devices can provide physiological and behavioral information, but long-term wearing may affect comfort and adherence [9,10]. Bed-based and mattress-integrated sensors, including pressure sensors, load sensors, piezoelectric films, bed-motion sensors, and accelerometers, are attractive for bedroom deployment because they can capture in-bed status, body movement, and bed-exit events without requiring participants to wear devices [11,12,18,20,21]. Contactless methods such as radar, Wi-Fi sensing, infrared arrays, and vision sensors can also support presence, posture, motion, or respiration sensing, but they may face challenges related to privacy, occlusion, deployment complexity, or environmental sensitivity [13,14,15]. Overall, most of these studies focus on physiological monitoring, sleep staging, posture, respiration, or sleep quality assessment, whereas bedroom environmental control requires state semantics that are directly linked to control needs.

Multimodal sleep monitoring systems further combine pressure, accelerometry, environmental sensing, radar, Wi-Fi, thermal, depth, or physiological signals to improve sleep assessment and state estimation [17,18,19,20]. These studies demonstrate the value of multimodal sensing, but their target outputs are usually sleep stages, sleep quality, respiration, posture, or clinical sleep-related indicators. In contrast, the present study focuses on control-oriented bedroom states that can support ventilation regulation, thermal preconditioning, night-light assistance, and low-disturbance actuator operation. Therefore, the proposed S0–S7 state system is not intended to replace PSG-based medical sleep staging, such as N1, N2, N3, or REM [16], but to provide a state sensing layer for smart bedroom environmental control.

Activity recognition in smart environments has also been widely studied in ambient intelligence and smart home research. For example, the SOFiA framework of De Carolis et al. learned and recognized routines and activities in a sensor-equipped smart office environment using workflow-based models [22]. Such studies highlight the importance of recognizing user routines and activities for adaptive intelligent environments. However, they are generally designed for daily activity or routine recognition in offices or homes, rather than for nocturnal bedroom-specific state recognition. The present study differs by focusing on human–bed interactions, bedside IEQ, room-context events, and time features during the sleep period.

Multimodal fusion methods have been widely used in human activity recognition, occupancy detection, and smart home monitoring [23,24,25,26]. Early fusion is simple but may ignore differences in modal importance and sensor quality, whereas late fusion may miss low-level complementary relationships among modalities [23,24]. Recent studies on gated fusion, cross-modal attention, hierarchical multimodal attention, and modality-dropout-based robustness have shown that adaptive fusion can regulate modality contributions, model cross-modal relevance, and reduce the effect of missing or degraded modalities [27,28,29,30]. For nocturnal bedroom state recognition, different states depend on different evidence: stable sleep-related states are mainly supported by bed-load stability and low bed-motion intensity; nocturnal out-of-bed events require joint evidence from bed-load reduction, PIR or door-contact events, and temporal context; and wake-up transition is associated with morning time, increased bed motion, and illuminance changes. These modality-dependent evidence patterns motivate the use of hierarchical and adaptive fusion rather than simple feature concatenation.

Smart bedroom state recognition also involves privacy protection, edge inference, and sensor node robustness. Bedrooms are highly privacy-sensitive spaces, and directly uploading raw behavioral data, audio, or video may reduce user acceptance [31]. Therefore, local preprocessing, feature construction, and state inference on an edge gateway are more suitable for sleep-related scenarios [32,33,34,35]. Practical deployment also needs to consider wireless packet loss, short-term missing data, node rebooting, time synchronization errors, and sensor drift. If a model is evaluated only on complete data, its deployment performance may be overestimated. Therefore, robustness to missing data, modality failures, sensing degradation, and edge inference overhead should be important components of sensor system evaluation [34,35]. The main characteristics, advantages, limitations, and roles of the sensing and recognition strategies discussed above are summarized in Table 1.

This comparison shows that a single sensing strategy is insufficient for control-oriented bedroom state recognition. Therefore, the proposed framework uses furniture-embedded bed-interaction sensing as the core evidence source and integrates bedside IEQ, room-context, and time features to recognize S0–S7 bedroom states.

Recent studies further demonstrate advances in unobtrusive bed sensing and multimodal sleep recognition [36,37]. Hoang et al. [36] used an accelerometric smart bed for contactless sleep position recognition, whereas Zhao et al. [37] developed adaptive multimodal fusion for sleep recognition. In contrast, the present study targets eight operational bedroom states by integrating bed interactions, bedside IEQ, room context, temporal cues, and sensing quality information. The contribution therefore lies in task-specific multimodal evidence organization, together with generalization, robustness, event, and edge deployment evaluation.

Overall, previous studies provide important foundations for sleep monitoring, bed-based sensing, activity recognition in smart environments, multimodal fusion, and edge intelligent sensing. However, three gaps remain. First, relatively few studies integrate furniture-embedded bed-interaction sensing with bedside IEQ and room-context sensing for bedroom-specific state recognition. Second, most existing sleep monitoring studies focus on sleep quality, sleep stages, posture, respiration, or physiological indicators rather than control-oriented bedroom state labels that reorganize occupancy, in-bed status, movement, bed-exit, and wake-up information for environmental control. Third, few studies jointly evaluate state recognition, event extraction, modality contribution, robustness to sensing degradation, edge deployment, and exploratory downstream application value. These gaps motivate the proposed bed-centered multimodal sensing framework and SA-HMF model.

To address these gaps, this study develops a privacy-preserving bed-centered multimodal bedroom sensing framework and a sleep-aware hierarchical multimodal fusion model, termed SA-HMF, for control-oriented bedroom state recognition. The framework integrates furniture-embedded bed-interaction sensing with bedside IEQ, room-context, and time features to recognize eight S0–S7 sleep-related bedroom states from window-level sensing data. The recognized states are further examined through exploratory downstream application analyses.

The main contributions are fourfold. First, a privacy-preserving bed-centered sensing framework integrates furniture-embedded bed-interaction sensing with bedside IEQ, room-context, and causal time features. Second, an operational S0–S7 state system reorganizes observable occupancy, in-bed, movement, bed-exit/return, and wake transition concepts according to bedroom control needs; it is not intended as a clinical sleep-stage taxonomy. Third, SA-HMF is developed as a bed-conditioned, quality-aware asymmetric hierarchical fusion architecture. Bed-load and bed-mounted accelerometry are first encoded into an intermediate bed-interaction representation, which then conditions the integration of IEQ, room-context, temporal, and sensing quality information. The contribution lies in this task-specific evidence organization rather than in a new generic attention or gating operator. Fourth, the framework is evaluated through controlled structural ablation, participant-level LOSO analysis, non-overlapping window sensitivity analysis, event extraction, sensing degradation robustness, edge deployment, and downstream application cases.

## 2. Materials and Methods

### 2.1. Study Framework and Experimental Bedroom Platform

This study addresses sleep-related state sensing in nocturnal bedroom environments and proposes a bed-centered multimodal bedroom sensing framework for recognizing sleep-related states oriented toward bedroom environmental control. The framework integrates furniture-embedded bed-interaction sensing with bedside IEQ sensing, room-context sensing, and time features. Unlike conventional room-level occupancy detection, the focus of this study is not simply to determine whether the bedroom is occupied but to identify fine-grained bedroom states that are directly relevant to environmental regulation, including unoccupied, occupied-out-of-bed, awake-in-bed, pre-sleep activity, stable sleep-related, movement/turning, nocturnal out-of-bed, and wake-up transition states. These states can provide a more semantically informative state sensing basis for subsequent ventilation regulation, thermal environment adjustment, night-light assistance, and low-disturbance control.

**Figure 1:**
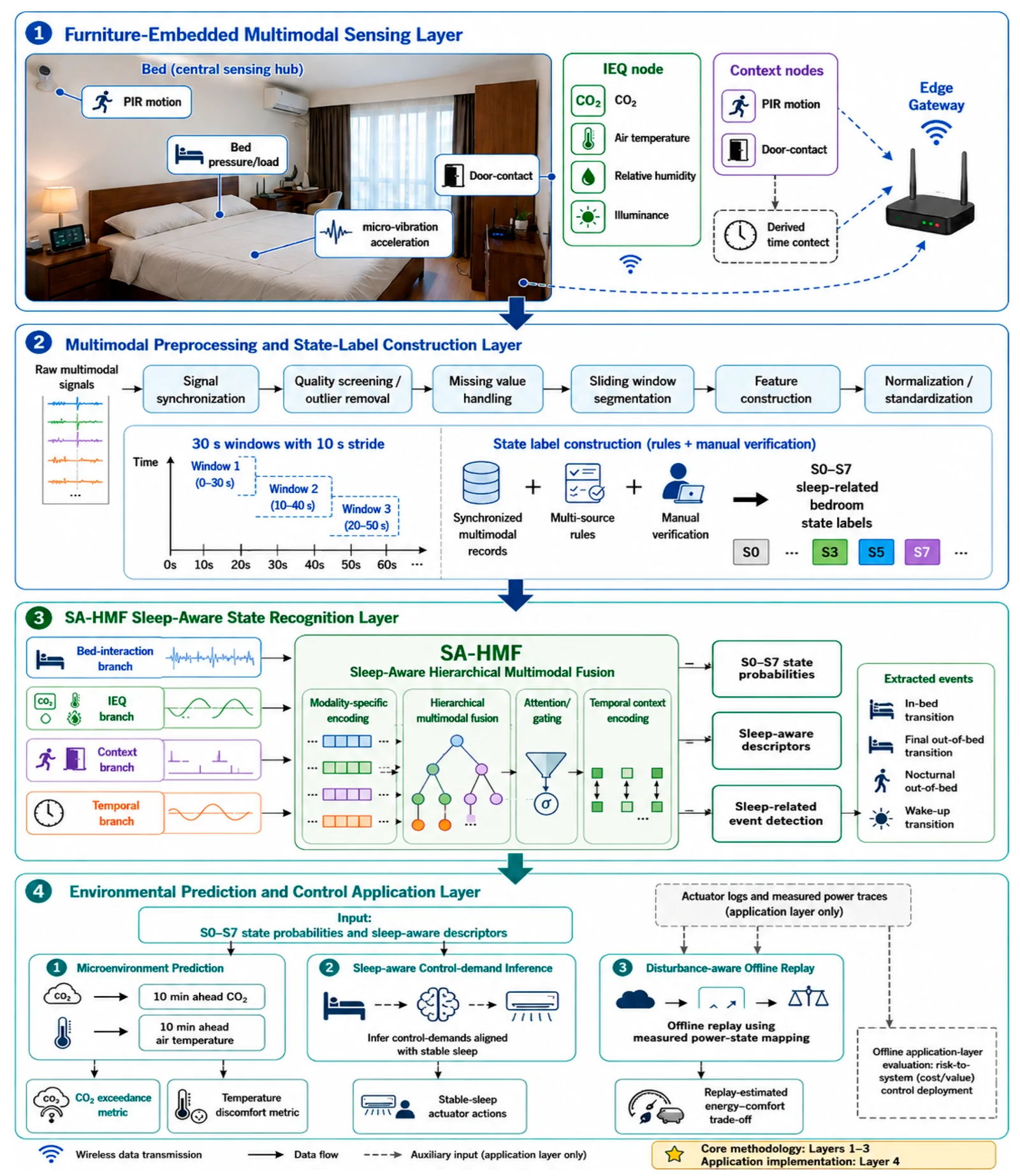
Overall workflow of the bed-centered multimodal sensing framework. The framework comprises four layers: multimodal sensing, preprocessing and state-label construction, SA-HMF state recognition, and downstream environmental prediction and control evaluation. The photograph illustrates the non-intrusive deployment of bed-interaction, bedside IEQ, and room-context nodes. Actuator and energy records are used only in the downstream application layer. No video or raw audio data were collected.

As shown in Figure 1, the proposed framework consists of four functional layers. The first layer is the furniture-embedded multimodal sensing layer, which collects human–bed interaction signals, bedside indoor environmental quality variables, and room-context events. The second layer is the multimodal preprocessing and state label construction layer, which performs temporal synchronization of multi-source sensing data, quality screening, sliding window segmentation, window-level feature extraction, and construction of control-oriented state labels. The third layer is the sleep-aware hierarchical multimodal fusion recognition layer, namely, the proposed SA-HMF model, which fuses bed-interaction, bedside environmental, room-context, and time features and outputs the probabilities of S0–S7 sleep-related bedroom states. The fourth layer is the environmental prediction and control application layer, which uses the recognized state probabilities as contextual inputs for microenvironment prediction, control-demand inference, and disturbance-aware offline application evaluation.

### 2.2. Multimodal Sensing System and Data Acquisition

**Figure 2:**
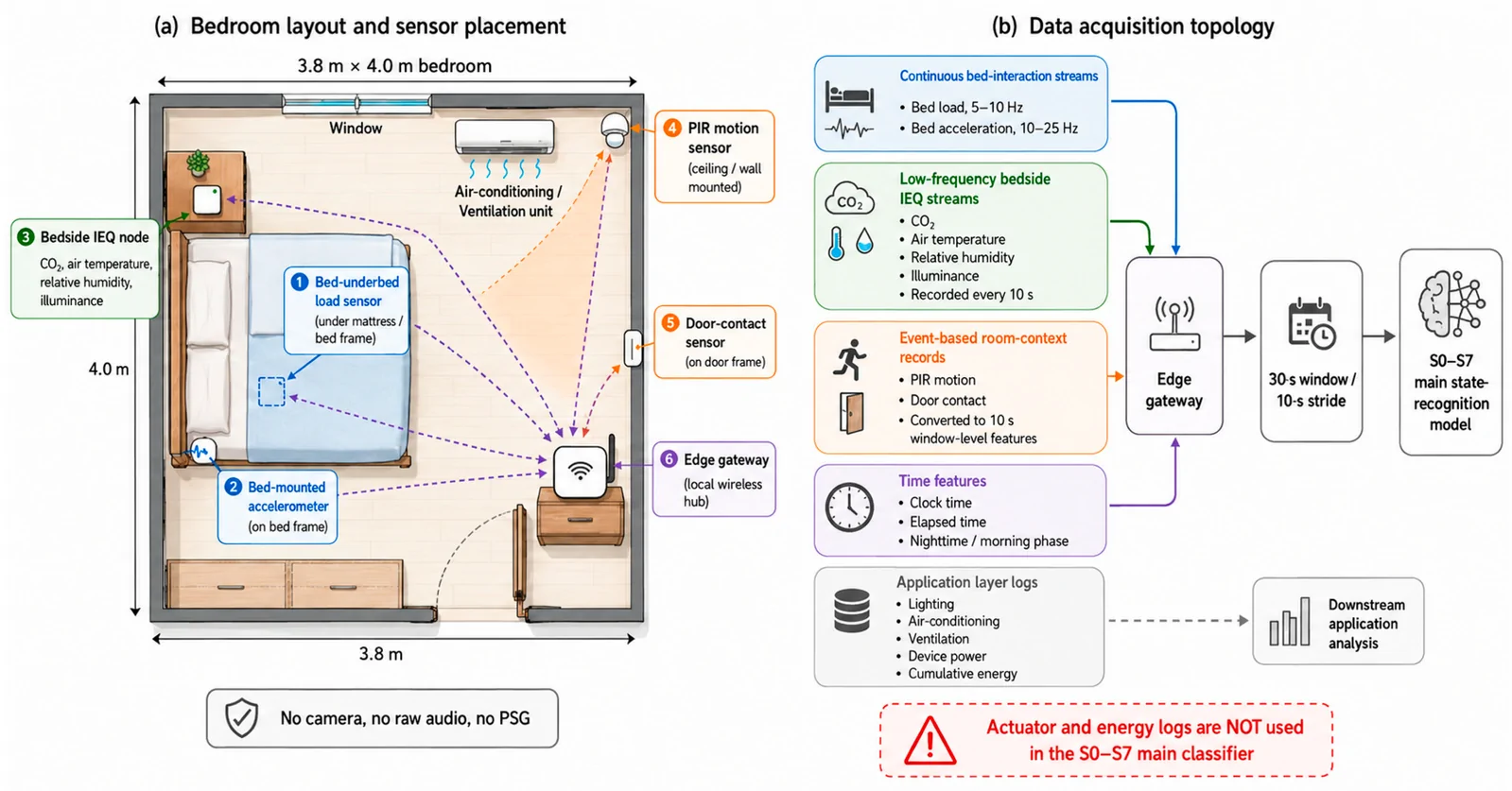
Experimental bedroom platform and sensor node deployment. (**a**) Layout of the experimental bedroom and non-intrusive sensor placement, including bed-integrated load sensing, bed-mounted accelerometry, bedside IEQ sensing, PIR motion sensing, door-contact sensing, and the local edge gateway. (**b**) Data acquisition topology for continuous sensing streams, event-based contextual records, time features, and application layer actuator/energy logs. Actuator and energy variables were recorded only for downstream application analysis and were not used as inputs to the S0–S7 main state recognition model.

The multimodal sensing system was configured as a bed-centered, non-intrusive bedroom sensing topology consisting of three categories of sensor nodes: bed-interaction nodes, bedside IEQ nodes, and room-context nodes. Bed-integrated load sensing (Interface, Inc., Scottsdale, AZ, USA) and bed-mounted accelerometry (TDK InvenSense, San Jose, CA, USA) captured direct human–bed interaction; bedside CO2, air temperature, and relative humidity sensing (Sensirion AG, Stäfa, Switzerland), together with illuminance sensing (ROHM Co., Ltd., Kyoto, Japan), described the near-bed microenvironment; and PIR motion sensing (Panasonic Industry Co., Ltd., Kadoma, Osaka, Japan) and door-contact sensing (OMRON Corporation, Kyoto, Japan) provided lightweight room activity and entry/exit cues. This topology was designed for control-oriented bedroom state recognition without using cameras, raw audio, or clinical PSG equipment. The sensor placement and data acquisition topology are shown in Figure 2.

Although the bed-integrated load sensor and the bed-mounted accelerometer were co-located on the same furniture item, they were not treated as two independent furniture modalities. Instead, they were grouped as one bed-interaction sensing branch because they captured complementary physical aspects of the same human–bed interaction process. The load sensor mainly described quasi-static bed occupancy, load transfer, and getting-into-bed or leaving-bed changes, whereas the accelerometer captured dynamic bed-motion patterns such as turning, body movement, and short unstable segments. Therefore, in this study, “furniture-embedded” specifically refers to the bed-interaction sensing branch, while the complete multimodal framework further integrates bedside IEQ and room-context sensing.

Lighting, air-conditioning, ventilation, and energy use records were collected as application layer logs. These variables were used only for downstream environmental prediction, control-demand inference, disturbance analysis, and offline replay evaluation and were not used as inputs to the S0–S7 main state recognition model.

The sensor deployment in the experimental bedroom followed three principles: proximity to the location where relevant behaviors occur, reduced privacy intrusion, and low deployment complexity. The bed-interaction nodes were installed in the bed structure or beneath the mattress to capture load variations and accelerometry-derived bed-motion patterns caused by getting into bed, leaving the bed, turning, and body movement. Compared with room-level occupancy sensors mounted on walls or ceilings, bed-interaction nodes were closer to the main location of nocturnal human–bed behaviors and were therefore particularly informative for identifying in-bed, leaving-bed, stable sleep-related, movement/turning, and nocturnal out-of-bed transition states. However, bed-interaction sensing alone was not assumed to be sufficient for all S0–S7 states; for prolonged absence from the bed, room-context and time features were also required to distinguish unoccupied, occupied-out-of-bed, and nocturnal out-of-bed states.

The bedside IEQ node was placed near the sleeping area, such as on a bedside table or at an appropriate height beside the bed, to measure environmental variables close to the occupant’s actual exposure level. The node was not placed directly near air-conditioning outlets, windows, door gaps, or strong light sources in order to reduce the influence of local airflow, local illumination, and short-term disturbances on the measurements. CO_2_ concentration was used to reflect ventilation- and occupancy-related changes, serving as an auxiliary cue for state recognition and as an input for ventilation-related prediction and demand inference in the application layer. Air temperature and relative humidity described the thermal and humidity environment, whereas illuminance helped distinguish pre-sleep activity, nocturnal out-of-bed, and wake-up transition states.

The room-context sensing nodes included a PIR motion sensor and a bedroom door-contact sensor. The PIR node detected human motion in the main activity area of the room and helped distinguish the unoccupied state from the occupied-out-of-bed state. The door-contact node was installed on the bedroom door to record entry, exit, nocturnal leaving, and return events. Because this study focused on control-oriented sleep-related bedroom state recognition rather than medical sleep diagnosis, high-privacy or high-complexity sensing devices were not used as core inputs.

The collected data included continuous sensing data, event-based contextual data, and window-level time features. Bed-load signals were sampled at relatively high rates to capture getting-into-bed, leaving-bed, and bed-load stability changes. Bed-mounted accelerometry was also sampled at relatively high rates to detect turning, body movement, and unstable sleep-related segments. Bedside IEQ variables changed more slowly and were therefore collected at lower sampling rates. PIR and door-contact sensors generated event-based records, including event timestamps and event states, which were later converted into window-level status indicators, event counts, or time-since-last-event features. Time features were generated using only the current window and previously observed information to represent pre-sleep, nighttime, and morning phases; future event times, such as final wake-up or final out-of-bed times, were not used as inputs to the main state recognition model.

To clarify the relative sampling requirements of different sensing streams, the data were organized at three recording levels. Bed-load signals were recorded at 5–10 Hz, and bed-mounted accelerometry was recorded at 10–25 Hz, because these channels were used to capture short-term bed-entry, bed-exit, turning, and body movement dynamics. Bedside IEQ variables, including CO_2_ concentration, air temperature, relative humidity, and illuminance, were recorded every 10 s because they changed more slowly. PIR and door-contact signals were event-based and were converted into 10 s window-level indicators, event counts, and time-since-last-event features during preprocessing. The complete retained variables and sampling or recording modes are provided in Supplementary Table S1.

A 30 s window length and a 10 s sliding stride were used for subsequent state recognition. This setting provided a balance between stable state recognition and sufficient temporal resolution for short-duration events such as nocturnal leaving-bed, returning-to-bed, body movement, and wake-up transitions. The detailed procedures for window segmentation, window-level feature extraction, handling missing data, and standardization are described in Section 2.4.

All sensing streams were synchronized through the local gateway and converted into window-level features during preprocessing. Each record retained timestamp and data quality information to support temporal alignment, missing data handling, and robustness analysis. Detailed sampling schemes, retained variables, gateway data fields, and application layer records are summarized in Supplementary Tables S1 and S2.

### 2.3. Experimental Protocol and Dataset Construction

An experimental dataset was constructed to evaluate S0–S7 bedroom state recognition, event extraction, generalization, robustness, and downstream application cases. This section reports the participants, recorded nights, experimental scenarios, and dataset construction. Temporal alignment, feature extraction, label construction, and missing data handling are described in Section 2.4.

#### 2.3.1. Participants, Recorded Nights, and Experimental Scenarios

The experiment was conducted in a typical bedroom with an area of 15.2 m^2^, a floor plan of 3.8 m × 4.0 m, and a ceiling height of 2.7 m. The room contained a bed, a bedside cabinet, lighting, air-conditioning or ventilation equipment, and standard bedroom furniture. Bed-interaction nodes, a bedside IEQ node, a PIR motion sensor, and a door-contact sensor were deployed non-intrusively according to the principles described in Section 2.2. Each nightly recording started when the participant entered the bedroom or began pre-sleep activity and continued until a short period after the participant finally left the bed and exited the bedroom the following morning. This recording range covered pre-sleep activity, awake-in-bed periods, main sleep periods, nocturnal out-of-bed episodes, morning wake-up transitions, and the unoccupied state after room exit.

Twenty healthy adults participated in the study, including 10 males and 10 females. Their ages ranged from 19 to 53 years, with a mean age of 31.8 ± 8.6 years. Each participant was scheduled for seven nights of recording, yielding 140 planned participant-nights. During data collection, 137 nights were successfully recorded; some nights were affected by incomplete device records, missing event logs, or abnormal key sensing modalities. After night-level and long-segment quality control, 127 valid participant-nights were retained, corresponding to 92.7% of the successfully recorded nights.

Table 2 summarizes the dataset construction. Both candidate windows and valid labeled windows were generated using a 30 s window length and a 10 s sliding stride. The stride-based equivalent coverage or duration in the table was calculated as the number of windows × 10 s/3600 and is reported only to describe the scale of overlapping window samples; it should not be interpreted as non-overlapping real recording duration. Thus, the participant number and valid participant-nights describe the experimental population and recording scale, whereas the number of labeled windows describes the overlapping window-level instances used for model training and evaluation.

To ensure that the dataset covered the key states required for bedroom environmental control, the recordings included not only natural sleep nights but also pre-sleep activity, nocturnal out-of-bed events, wake-up transitions, and environmental variations. Most nights followed the participants’ natural sleep habits, without enforcing specific bedtimes, wake-up times, or sleeping postures. A small number of nights included mild guidance to increase the coverage of pre-sleep activity, wake-up transitions, or environmental changes. Sensor missingness and node failure were not introduced as field interventions, but they were simulated offline in later robustness experiments. The experimental scenarios, their purposes, and the corresponding data sources and implementation procedures are summarized in Table 3.

A nocturnal out-of-bed event was defined as a complete episode in which the participant left the bed after a sleep-related or movement state and returned to the bed after a period of time. Such events were typically characterized by a marked reduction in bed load together with PIR or door-contact events. A wake-up transition was defined as a continuous morning process in which the participant gradually shifted from a stable sleep-related or movement state to wakefulness and finally left the bed or the bedroom. Because a wake-up transition is not an instantaneous event, its labeling and evaluation were based on continuous windows or time intervals rather than a single time point.

#### 2.3.2. Night-, Long-Segment-, and Window-Level Quality Control

Three levels of quality control were applied before model training: night-level screening, long-segment-level screening, and window-level screening. Night-level screening excluded recordings with whole-night failure of key sensors, severe synchronization problems, missing key event logs, or unreliable main state transition information. Long-segment-level screening removed prolonged invalid segments within otherwise usable nights. Window-level screening further excluded samples with severe missingness in key modalities, obvious abnormal values, communication failures, or uncertain state labels after manual verification.

After night-level and long-segment-level screening, 127 valid participant-nights were retained. These synchronized segments generated 365,921 candidate windows using a 30 s window and a 10 s stride. Window-level screening removed 23,237 windows, accounting for 6.35% of the candidate windows. The final dataset contained 342,684 valid labeled windows for model training, validation, and testing. Detailed quality control criteria and dataset construction statistics are summarized in Table 2 and Supplementary Table S8.

### 2.4. Preprocessing, Feature Extraction, and State Label Construction

Because the experimental bedroom platform collected bed-interaction signals, bedside IEQ variables, room-context events, actuator logs, energy-related records, and time features simultaneously, the modalities differed substantially in sampling frequency, data type, and event granularity. Therefore, before the data were fed into the SA-HMF model, multi-source data were processed through unified temporal alignment, quality screening, window segmentation, feature extraction, and state label construction.

Based on the valid recording segments obtained in the previous section, this section further describes how multimodal data were converted into window-level training samples and defines the control-oriented S0–S7 sleep-related bedroom state label system used in this study. The preprocessing and label construction procedures in this section served the S0–S7 main state recognition task.

**Figure 3:**
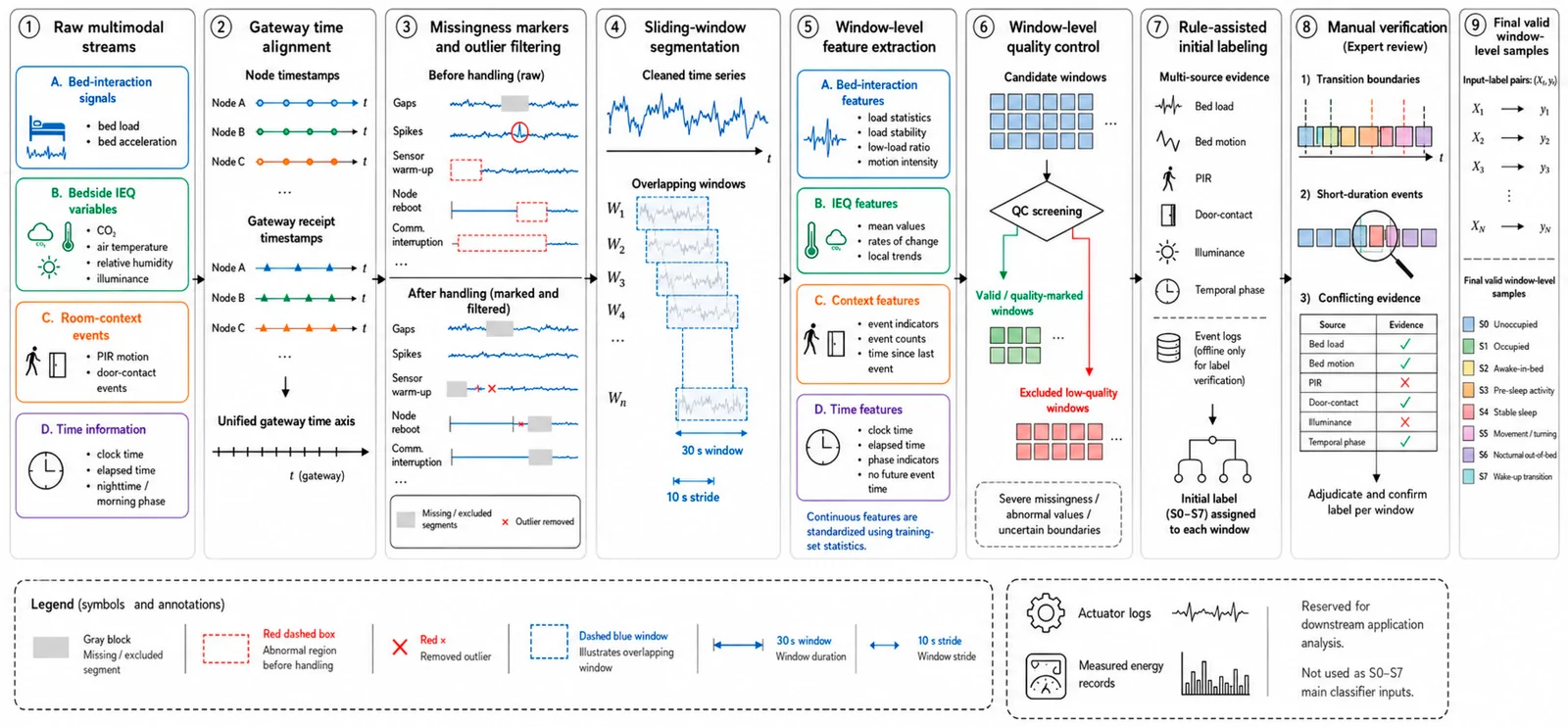
Multimodal preprocessing, quality control, and S0–S7 label construction pipeline.

The complete preprocessing, quality control, and S0–S7 label construction pipeline is illustrated in Figure 3.

#### 2.4.1. Temporal Alignment, Quality Screening, and Window-Level Feature Extraction

All sensing records contained both node timestamps and gateway receipt timestamps. To ensure that different modalities could be fused on a unified time axis, gateway time was used as the reference time axis to align bed-load signals, bed-mounted accelerometry, CO_2_ concentration, air temperature, relative humidity, illuminance, PIR events, door-contact events, and actuator event logs. For continuous high-frequency data, including bed-load signals and bed-mounted accelerometry, the original time series were retained and aggregated into statistical features during windowing. For low-frequency IEQ data, including CO_2_ concentration, air temperature, relative humidity, and illuminance, measurements were mapped to the corresponding windows according to their timestamps, and window-level means, rates of change, and trend features were extracted. For event-based data, including PIR and door-contact events, event timestamps were converted into window-level event indicators, event counts, and time-since-last-event features. State changes of lighting, air-conditioning, and ventilation devices were also recorded on the same time axis but were converted only into application layer variables and were not used as inputs to the S0–S7 main state recognition model.

Data quality screening included outlier detection, short-term missingness marking, removal of long missing segments, and exclusion of segments with uncertain labels [38]. For bed-load signals and bed-mounted accelerometry, outliers mainly included abrupt values beyond the reasonable sensing range, continuous zero-value segments caused by node rebooting, and continuous missing data caused by communication interruption. For IEQ data, outliers mainly included readings during CO_2_ sensor warm-up, abnormal short-term jumps in air temperature or relative humidity, and extreme values caused by illuminance sensor obstruction. For event-based data, including PIR and door-contact events, the main checks included whole-night non-response, abnormal timestamps, discontinuous event states, and missing event records.

Short-term missing data were not removed directly. Instead, missingness was first retained as an explicit marker and then handled according to the variable type and missing duration. For low-frequency IEQ variables, including CO_2_ concentration, air temperature, relative humidity, and illuminance, short missing periods were filled using neighboring valid values or window-level interpolation while the missingness marker was retained. For key high-frequency modalities, including bed-load signals and bed-mounted accelerometry, if missingness occurred near important state transitions such as getting into bed, leaving bed, nocturnal out-of-bed event, or wake-up transition, the corresponding windows were marked as low-quality windows. If a window contained severe missingness in key modalities, or if the state boundary could not be confirmed using multi-source information, the window was excluded from the final training set.

A fixed-length sliding window was used to segment the synchronized multimodal data. The window length was set to 30 s, and the sliding stride was set to 10 s. Let denote the start time of the i-th window. The t-th window was defined as:

$$\left.W_{t}=[\tau_{t},\tau_{t}+L\right)$$

where L=30 s denotes the window length, and the stride between adjacent windows was 10 s. This setting provided a balance between stable state recognition and sufficient temporal resolution for short-duration events, such as nocturnal leaving-bed, returning-to-bed, body movement, and wake-up transition states.

For each window, window-level features were extracted from different modalities. Bed-load features mainly included the window mean, standard deviation, maximum, minimum, load variation amplitude, load stability, low-load ratio, and in-bed indicator features. Accelerometry-derived bed-motion features mainly included the acceleration root mean square, variance, peak count, body movement intensity, and short-term bed-motion variation features. These features were used to characterize bed-interaction states, such as in-bed, out-of-bed, turning, body movement, and stable sleep-related states.

Bedside IEQ features included the window-level means, rates of change, and local trends of CO_2_ concentration, air temperature, relative humidity, and illuminance. CO_2_ dynamics reflected ventilation- and occupancy-related changes; air temperature and relative humidity described the thermal and humidity environment; and illuminance changes helped distinguish pre-sleep activity, nocturnal out-of-bed, and morning wake-up transition states. Contextual events, including PIR and door-contact events, were converted into within-window event indicators, event counts, event duration states, and the time since the most recent event. Time features included clock time, elapsed time after getting into bed or lights-off, time since the most recent observed bed-interaction or contextual event, and nighttime/morning phase indicators. All time features were constructed using only information available in the current window and previous windows; future event information, such as final wake-up time or final out-of-bed time, was not used as model input.

Continuous variables were standardized before model training. For a continuous feature x in the training set, normalization was performed using the training set mean and standard deviation:

$$\hat{x}=\frac{x-\mu_{train}}{\sigma_{train}}$$

where $\mu_{train}$ and $\sigma_{train}$ denote the mean and standard deviation of the feature in the training set, respectively. The validation and test sets were standardized using the statistics computed from the training set to avoid data leakage. Event-based variables were represented using binary encoding, event counts, or time-since-last-event features. Modality availability and missingness status were retained as quality markers for subsequent gated fusion and robustness analysis.

#### 2.4.2. Separation Between Main State Recognition Inputs and Application Layer Inputs

After temporal alignment and feature extraction, each window was represented by four groups of inputs for the S0–S7 main state recognition task:

X_i_ = {X_i_^bed^, X_i_^IE^Q, X_i_^ctx^, X_i_^time^},

where X_i_^bed^ denotes bed-load and accelerometry-derived bed-motion features, X_i_^IE^Q denotes bedside environmental features, X_i_^ctx^ denotes PIR and door-contact contextual features, and X_i_^time^ denotes time-related features. These four feature groups were the only inputs to the SA-HMF main classifier.

Actuator logs and energy-related variables, including lighting status, air-conditioning status, ventilation status, device power, and accumulated electricity use, were excluded from the S0–S7 classifier. They were used only in the downstream application layer after SA-HMF state probabilities had been obtained. This separation prevented device actions or energy use patterns from leaking into the state recognition task.

Post hoc information, such as final wake-up time, final out-of-bed time, and nocturnal return-to-bed events, was used only for offline label verification, state boundary confirmation, and event-level evaluation. It was not used as input for real-time state recognition. The complete variable separation is summarized in Supplementary Table S2.

#### 2.4.3. Control-Oriented S0–S7 State System

This study defines an operational state system for bedroom environmental prediction and control. For each window i, the corresponding state label is denoted as:

$$y_{t}\in \{S_{0},S_{1},S_{2},S_{3},S_{4},S_{5},S_{6},S_{7}\}$$

The S0–S7 scheme is an operational, control-oriented state system rather than a clinical sleep-stage taxonomy. It reorganizes observable room occupancy, in-bed, stable resting, movement, bed-exit/return, and wake transition concepts according to their implications for bedroom environmental control. The eight-state partition was developed for the sensing configuration and application objectives of this study. It is not intended to infer PSG-based N1, N2, N3, or REM stages.

Table 4 summarizes the definitions, main labeling evidence, and control implications of the S0–S7 state system used in this study.

Here, $S_{6}$ nocturnal out-of-bed event does not refer only to the instant of leaving bed but to the complete event segment from leaving bed at night to returning to bed. This definition is more suitable for environmental control scenarios because nocturnal out-of-bed episodes often require dim lighting or safety assistance for a certain duration, rather than a control response triggered only at the moment of bed exit.

Similarly, $S_{7}$ wake-up transition is not defined as a single time point. Instead, it represents a continuous morning transition process in which the occupant gradually shifts from a stable sleep-related or movement state to wakefulness and eventually leaves the bed or the bedroom. Defining wake-up transition as a sustained state can support applications such as gradual lighting, thermal preconditioning, and ventilation preconditioning. This label design is better aligned with bedroom environmental control requirements because control systems generally need to recognize sustained state processes rather than only instantaneous actions.

The complete S0–S7 system has not been externally validated across independent bedrooms, bed types, sensing installations, or populations. The present study therefore evaluates its operational usefulness within the tested framework rather than claiming universal validity of the state number or boundaries.

#### 2.4.4. Rule-Assisted Initial Labeling and Manual Verification

The S0–S7 state labels were generated using a combination of multi-source rule-assisted initial labeling and manual verification. This procedure did not rely solely on subjective manual judgment or on a single threshold-based rule. Instead, initial labels were first assigned according to bed-interaction signals, room-context events, illuminance, temporal phase, and participant event logs. State boundaries, short-duration events, and segments with conflicting rule outputs were then manually verified.

The initial labeling process mainly used bed-load signals, bed-mounted accelerometry, PIR events, door-contact events, illuminance changes, time features, and participant event logs. Bed-load signals were used to preliminarily determine in-bed and out-of-bed states. Bed-mounted accelerometry was used to identify body movement, turning, and unstable sleep-related segments. PIR and door-contact events were used to support the identification of occupied-out-of-bed states, bedroom entry, bedroom exit, nocturnal leaving-room events, and return-to-room events. Illuminance and time features were used to help distinguish pre-sleep activity, nocturnal out-of-bed episodes, and wake-up transitions. Participant event logs mainly included key time points such as bedroom entry, getting into bed, lights-off, preparing to sleep, nocturnal out-of-bed events, return to bed, wake-up, final out-of-bed event, and bedroom exit. These event logs were used only for offline label verification and boundary confirmation and were not used as inputs to the SA-HMF main state recognition model.

Specifically, rule-assisted initial labeling was conducted in three steps. First, in-bed, out-of-bed, stable, and movement segments were preliminarily identified according to bed-load thresholds and bed-motion intensity. A sustained bed-load value above the in-bed threshold was used to indicate an in-bed state. A marked and sustained reduction in bed-load below the out-of-bed threshold was used to indicate an out-of-bed state. If accelerometry-derived bed-motion intensity or peak activity increased markedly while the occupant remained in bed, the corresponding segment was initially labeled as movement or turning.

Second, the initial labels were contextually refined using PIR events, door-contact events, illuminance, and temporal phase. For example, when the bed-load signal indicated an out-of-bed state, PIR activity was present, and the segment occurred during a normal pre-sleep or post-wake period, the window was more likely to be labeled as $S_{1}$ occupied-out-of-bed. When the same out-of-bed pattern occurred after the main nocturnal sleep period and was followed by a return-to-bed event, it was labeled as $S_{6}$ nocturnal out-of-bed. To distinguish $S_{2}$ awake-in-bed from $S_{3}$ pre-sleep activity states, the system jointly considered bed-motion intensity, illuminance level, and the pre-sleep temporal phase. For $S_{7}$ wake-up transition, the label was confirmed using the morning time period, increased bed motion, illuminance changes, and the subsequent final out-of-bed event.

Third, manual verification was performed for state transition boundaries, short-duration events, and segments with conflicting rule outputs. Manual verification focused on four types of cases: unclear boundaries of getting into bed, leaving bed, returning to bed, and wake-up transition; short-duration body movement, turning, or temporary out-of-bed segments that could easily be assigned to other states; conflicts among bed-interaction signals, PIR events, door-contact events, illuminance, and event logs; and segments in which short-term missingness in key modalities or incomplete event logs prevented reliable rule-based labeling. Windows whose labels remained uncertain after manual verification were not forced into any class and were excluded during quality control.

The natural imbalance of nocturnal bedroom behaviors was preserved. Stable sleep-related states typically occupied most of the nighttime period, whereas nocturnal out-of-bed and wake-up transition states were low-frequency but control-relevant key states. Preserving this natural class imbalance enabled a more realistic evaluation of model performance in practical bedroom environments. During model training and evaluation, class weights and metrics such as macro-F1 were used to address class imbalance.

Through this procedure, each valid window was finally represented as a main state-recognition input–label pair:

$$\left(X_{t}^{state},y_{t}\right)$$

where

$$X_{t}^{state}=\left[X_{bed,t},X_{IEQ,t},X_{ctx,t},X_{time,t}\right]$$

denotes the window-level multimodal features used for SA-HMF main state recognition and $y_{t}$ denotes the corresponding S0–S7 control-oriented sleep-related bedroom state label. This representation provided the unified input–label basis for model training, validation, robustness analysis, and edge implementation.

### 2.5. Sleep-Aware Hierarchical Multimodal Fusion Model

SA-HMF is a bed-conditioned, quality-aware asymmetric hierarchical fusion architecture. Attention, gating, and hierarchical encoding are established techniques; the contribution lies in organizing them according to the physical evidence structure of nocturnal bedroom states. Bed-load and bed-mounted accelerometry are first encoded into a bed-interaction representation describing occupancy, movement intensity, and load stability. This representation then conditions the weighting of IEQ, room-context, and temporal features, while sensing-quality markers regulate degraded or unavailable inputs.

**Figure 4:**
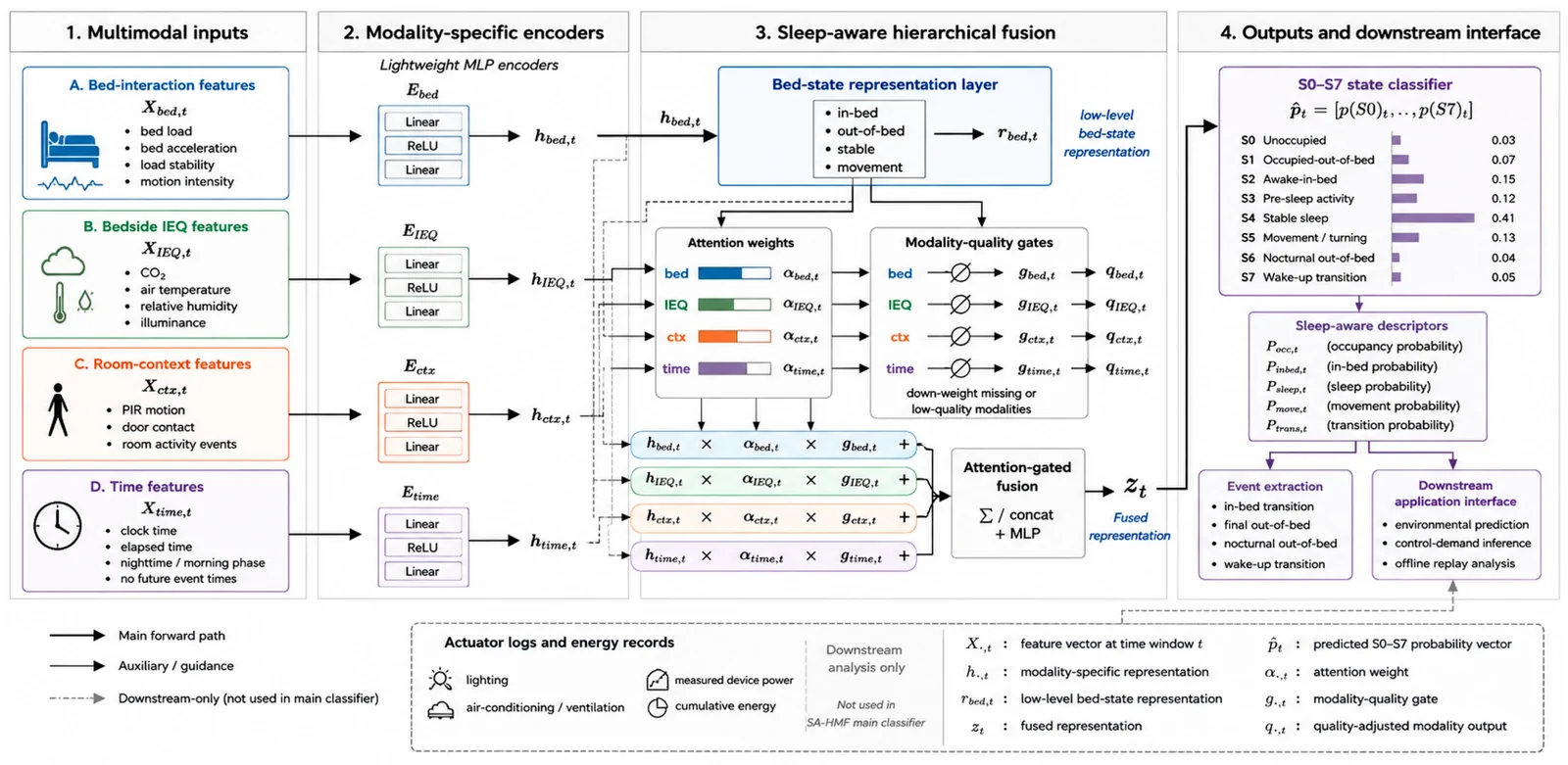
Architecture of the sleep-aware hierarchical multimodal fusion model.

The architecture examines three task-specific assumptions: bed interaction provides the primary physical anchor; contextual evidence should be interpreted conditionally on bed interaction; and modality contribution should depend on both relevance and sensing quality. These assumptions are evaluated through hierarchy removal, attention/gating removal, modality ablation, and sensing degradation analysis. The architecture is shown in Figure 4.

#### 2.5.1. Hierarchical Architecture and Modality Encoders

The classifier contains two asymmetrically organized levels: an intermediate bed-interaction representation and a final bedroom state classifier. The first summarizes direct human–bed interaction; the second uses it as a conditioning signal when integrating IEQ, room-context, time, and sensing quality information for S0–S7 prediction.

For the t-th time window, the input to the SA-HMF main state recognition model is defined as:

$$X_{t}^{state}=[X_{bed,t},X_{IEQ,t},X_{ctx,t},X_{time,t}]$$

where $X_{bed,t}$ denotes bed-interaction features extracted from bed-load sensing and bed-mounted accelerometry; $X_{IEQ,t}$ denotes bedside environmental features, including CO_2_ concentration, air temperature, relative humidity, and illuminance; $X_{ctx,t}$ denotes contextual features, including PIR events, door-contact events, and bedroom entry/exit events; and $X_{time,t}$ denotes time features, including clock time, elapsed time after getting into bed or lights-off, time since the most recent observed event, and nighttime/morning phase indicators.

Because different modalities have different data types and physical meanings, SA-HMF uses a lightweight encoder for each modality. For modality m, the window-level representation is encoded as:

$$h_{t}^{m}=f_{m}(X_{t}^{m}),m\in \{bed,IEQ,ctx,time\}$$

where $X_{t}^{m}$ is the input feature vector of modality m, $f_{m}(\cdot )$ is the corresponding modality encoder, and $h_{t}^{m}$ is the hidden representation. A lightweight multilayer perceptron (MLP) is used as the modality encoder because each window has already been represented by statistical, event-based, and temporal features after preprocessing. This design reduces model complexity while retaining sufficient representational capacity for edge deployment.

A single modality encoder can be written as:

$$h_{t}^{m}=\sigma \left(W_{2}^{m}\sigma (W_{1}^{m}X_{t}^{m}+b_{1}^{m})+b_{2}^{m}\right)$$

where $W_{1}^{m}$, $W_{2}^{m}$, $b_{1}^{m}$, and $b_{2}^{m}$ are learnable parameters of the modality encoder and σ(·) denotes a nonlinear activation function. Dropout or layer normalization can be added to reduce overfitting. The outputs of all modality encoders are mapped to a shared dimension for subsequent multimodal fusion.

Specifically, the bed encoder $f_{bed}(\cdot )$ processes bed-load, bed-motion, load stability, and movement intensity features to extract bed-interaction representations. The IEQ encoder $f_{IEQ}(\cdot )$ processes CO_2_ concentration, air temperature, relative humidity, and illuminance to represent near-bed microenvironmental changes. The context encoder $f_{ctx}(\cdot )$ processes PIR, door-contact, and bedroom entry/exit events to represent room activity and state transitions. The time encoder $f_{time}(\cdot )$ processes clock time, elapsed time after getting into bed or lights-off, and nighttime/morning phase indicators to support the distinction among pre-sleep, nighttime, and wake-up transition states.

The bed state representation layer first generates the bed-interaction representation:

$$h_{bed,t}=f_{bed}(X_{bed,t})$$

and can further estimate intermediate bed state probabilities:

$$p_{bed,t}=softmax(W_{bed}h_{bed,t}+b_{bed})$$

where $p_{i}^{bed}$ denotes intermediate descriptors related to in-bed/out-of-bed status, movement, and stability. This representation neither replaces the final S0–S7 output nor represents clinical sleep staging. Its function is to provide a physical anchor before environmental, contextual, and temporal evidence is interpreted. The complete architecture is summarized in Supplementary Table S3.

#### 2.5.2. Attention-Gated Multimodal Fusion

Different modalities contribute differently to different sleep-related bedroom states. Bed-load and accelerometry-derived bed-motion features are most important for recognizing in-bed, out-of-bed, stable sleep-related, and movement/turning states. PIR and door-contact events are useful for identifying unoccupied, occupied-out-of-bed, nocturnal leaving-room, and return-to-room events. Illuminance and time features help distinguish pre-sleep activity and wake-up transition. IEQ variables are not always dominant for state classification, but they provide useful context for occupancy changes, illuminance changes, microenvironment prediction, and downstream application analysis. Therefore, consistent with previous studies on gated, cross-modal attention-based, and hierarchical multimodal fusion [27,28,29], SA-HMF adopts an attention-gated multimodal fusion strategy rather than simple feature concatenation.

Attention-based multimodal fusion has been used to assign adaptive weights to modalities according to state context and cross-modal relevance [28,29]. First, the model computes an attention score for each modality using the current modality representation and the bed-interaction representation:

$$e_{t}^{m}=v^{⊤}tanh(W_{h}h_{t}^{m}+W_{b}h_{bed,t}+b_{h})$$

The normalized modality weight is then obtained by softmax:

$$\alpha_{t}^{m}=\frac{exp(e_{t}^{m})}{\sum_{k}exp(e_{t}^{k})}$$

where $e_{t}^{m}$ is the attention score of modality m in window t, $\alpha_{t}^{m}$ is the corresponding normalized modality weight, and $h_{bed,t}$ is the bed-interaction representation generated by the bed state representation layer. By incorporating the bed representation, the model can prioritize modalities that are more relevant to human–bed interactions under the current state. For example, when the bed-load pattern is stable and bed motion is low, the model can increase the contribution of the bed modality to stable sleep-related state recognition. When bed load decreases and PIR or door-contact events occur, the model can increase the contribution of contextual features to out-of-bed or nocturnal out-of-bed recognition.

Second, following gated multimodal fusion, modality dropout strategies, and robustness-aware sensing-model design [27,30,39], a modality gating mechanism is introduced to improve robustness to missing data, short-term node failure, and modality quality degradation. Let $r_{t}^{m}$ denote the data quality or availability marker of modality m in window t. The gate value is defined as:

$$g_{t}^{m}=\sigma (W_{g}[h_{t}^{m},r_{t}^{m}]+b_{g})$$

where $g_{t}^{m}$ represents the effective contribution of modality m in the current window. If a modality is missing, abnormal, or marked as low-quality, the gate reduces its contribution to the fused representation. If the modality is of high quality, the model retains its contribution to state recognition.

The modality representation after attention and gating is:

$${\tilde{h}}_{t}^{m}=\alpha_{t}^{m}\cdot g_{t}^{m}\cdot h_{t}^{m}$$

The final fused representation is:

$$h_{t}=\phi ([{\tilde{h}}_{t}^{bed},{\tilde{h}}_{t}^{IEQ},{\tilde{h}}_{t}^{ctx},{\tilde{h}}_{t}^{time}])$$

where ϕ(·) denotes the fusion layer, implemented using fully connected layers and nonlinear activation functions. This fusion structure enables SA-HMF to adaptively adjust modality contributions according to both state context and data quality. When the bed node is available, the model can rely more on bed-interaction signals to recognize in-bed, out-of-bed, and movement states. When context nodes are temporarily missing, the model can still use bed, IEQ, and time features to maintain basic recognition performance. When IEQ variables are briefly missing, the missingness marker and gating mechanism can reduce their negative influence on classification. Thus, attention-gated fusion supports both multimodal state recognition and missing data robustness.

#### 2.5.3. State Probability Output and Downstream State Descriptors

The core output of the SA-HMF main classifier is the S0–S7 state probability vector for each time window, rather than specific control demands. Using the fused representation $h_{t}$, the main classifier outputs:

$${\hat{p}}_{t}=softmax(W_{state}h_{t}+b_{state})$$

where

$${\hat{p}}_{t}=[{\hat{p}}_{t,0},{\hat{p}}_{t,1},\ldots ,{\hat{p}}_{t,7}]$$

and ${\hat{p}}_{t,k}$ denotes the predicted probability that window t belongs to state $S_{k}$. The final predicted label is:

$${\hat{y}}_{t}=S_{arg\underset{k\in \{0,\ldots ,7\}}{max}{\hat{p}}_{t,k}}$$

where $S_{k}$ denotes one of the eight control-oriented sleep-related bedroom states. The predicted state labels are used for window-level state recognition evaluation, confusion analysis, key event detection, cross-night and cross-subject generalization analysis, and edge deployment testing.

To connect the main state recognition model with downstream environmental prediction and control applications, a set of sleep-aware state descriptors is further derived from ${\hat{p}}_{t}$:

$$d_{t}^{state}=[P_{t}^{occ},P_{t}^{bed},P_{t}^{sleep},P_{t}^{move},P_{t}^{trans}]$$

where $P_{t}^{occ}$, $P_{t}^{bed}$, $P_{t}^{sleep}$, $P_{t}^{move}$, and $P_{t}^{trans}$ denote the probabilities related to room occupancy, in-bed status, stable sleep, movement/turning, and nocturnal out-of-bed or wake-up transition states, respectively. These descriptors are derived from the S0–S7 state probabilities or from bed/context information in the main state recognition inputs, without introducing actuator logs or energy records.

In a simplified form, the room occupancy probability is:

$$P_{t}^{occ}=1-{\hat{p}}_{t,0}$$

The in-bed probability is computed from awake-in-bed, pre-sleep activity, stable sleep, movement/turning, and wake-up transition states:

$$P_{t}^{bed}={\hat{p}}_{t,2}+{\hat{p}}_{t,3}+{\hat{p}}_{t,4}+{\hat{p}}_{t,5}+{\hat{p}}_{t,7}$$

The stable-sleep and movement probabilities are:

$$P_{t}^{sleep}={\hat{p}}_{t,4}$$

$$P_{t}^{move}={\hat{p}}_{t,5}$$

The transition-related probability is:

$$P_{t}^{trans}={\hat{p}}_{t,6}+{\hat{p}}_{t,7}$$

These derived descriptors link the S0–S7 main state recognition output to downstream environmental prediction and control-related application tasks, but they are not equivalent to control commands. The complete definitions, sources, and relationships between these descriptors and the SA-HMF main classifier are provided in Supplementary Table S4. Ventilation demand, thermal regulation demand, lighting demand, and actuator disturbance risk are generated by the application layer modules using SA-HMF state outputs, IEQ variables, actuator logs, and measured energy records as inputs.

### 2.6. Model Training, Validation, and Edge Implementation

To evaluate the effectiveness, generalization ability, event detection capability, and deployment feasibility of SA-HMF for sleep-related bedroom state recognition, the experimental design included model training, data splitting, event postprocessing, and edge implementation. Model training addressed multimodal feature standardization, class imbalance, loss construction, and hyperparameter settings. Model validation used a random window split, a night-independent split, a subject-independent split, and leave-one-subject-out cross-validation to evaluate performance under different generalization conditions. Event postprocessing converted window-level S0–S7 state probability sequences into in-bed transition, final out-of-bed transition, nocturnal out-of-bed, and wake-up transition events. Edge implementation evaluated whether the trained model could run in real time on a local low-power bedroom gateway.

#### 2.6.1. Training Strategy

SA-HMF was trained using the window-level input–label pairs defined in Section 2.4.2. All continuous features were standardized using training set statistics, and the same statistics were applied to the validation and test sets to avoid data leakage. Event-based and time-related variables were encoded as binary indicators, event counts, time-since-last-event features, or phase indicators, as described in Section 2.4.1.

Because nocturnal bedroom states were naturally imbalanced, class weights were introduced during training. In the dataset, $S_{4}$ stable sleep was the majority class, whereas $S_{6}$ nocturnal out-of-bed event was a low-frequency but control-relevant key state. Directly using ordinary cross-entropy could bias the model toward majority states. Therefore, class weights $w_{c}$ were calculated from the class distribution in the training set, and weighted cross-entropy was used as the S0–S7 state recognition loss:

$$L_{state}=-\frac{1}{B}\sum_{t=1}^{B}\sum_{c=0}^{7}w_{c}y_{t,c}log({\hat{p}}_{t,c})$$

where B is the batch size, $y_{t,c}$ indicates whether window t belongs to class c, ${\hat{p}}_{t,c}$ is the predicted probability of class c, and $w_{c}$ is the class weight. Class weights were computed only from the training set, without using validation or test information.

The main classifier was trained using the weighted cross-entropy loss defined above.

The recognition inputs followed the separation defined in Section 2.4.2. Event detection was performed by postprocessing the predicted state probability sequence, whereas environmental prediction and control-demand inference were evaluated separately in the application layer.

A lightweight training strategy was adopted to balance recognition performance and edge deployment feasibility. The AdamW optimizer was used with an initial learning rate of $1\times 10^{-3}$, weight decay of $1\times 10^{-4}$, batch size of 256, and a maximum of 100 epochs. Validation Macro-F1 was used as the main early stopping criterion. Training stopped if validation Macro-F1 did not improve for 10 consecutive epochs, and the model parameters with the best validation performance were retained. Dropout with a rate of 0.2 was used to reduce overfitting. The random seed was fixed at 42 for all experiments to improve reproducibility. Model implementation was performed using Python 3.11.9 and PyTorch 2.5.1; the LightGBM baseline used LightGBM 4.5.0.

#### 2.6.2. Data Splitting, Validation Scope, and Window Overlap Sensitivity

Because adjacent 30 s windows generated with a 10 s stride shared 20 s of data, the 342,684 windows were treated as correlated model instances rather than independent observations. Random splitting could place highly similar neighboring windows in both training and test sets; therefore, the subject-independent split was used as the primary protocol, with participants treated as the independent units for generalization analysis.

For the subject-independent split, 14 participants were used for training, three for validation, and three for testing. No test participant data were used for standardization, class weighting, model selection, or early stopping. This protocol evaluated transfer to unseen participants within the tested bedroom and sensing configuration.

The night-independent split assigned different nights from the same participants to the training, validation, and test sets and therefore evaluated cross-night stability within the same setting. The random window split used a 70%/15%/15% ratio and was retained only as a potentially optimistic reference.

Twenty-fold LOSO evaluation characterized participant-level variability within the tested setting. In each fold, one participant was held out for testing. Subject-independent and LOSO evaluation do not constitute cross-bedroom, cross-bed, or cross-population validation.

A non-overlapping window sensitivity analysis was additionally conducted using the same 30 s window length and a 30 s stride. The participant split, features, preprocessing, architecture, class weighting, and training settings were unchanged. Full SA-HMF and the non-hierarchical variant were retrained to assess whether the primary findings depended on adjacent window overlap.

#### 2.6.3. Event Extraction from State Probability Sequences

The basic output of SA-HMF is the window-level S0–S7 state probability vector, while key sleep-related events need to be extracted from continuous probability sequences. The trained SA-HMF output sequence was converted into four event types: in-bed transition, final out-of-bed transition, nocturnal out-of-bed event, and wake-up transition. This step was a postprocessing procedure and did not change the training objective of the SA-HMF main classifier.

For the t-th window, the SA-HMF output was:

$${\hat{p}}_{t}=[{\hat{p}}_{t,0},{\hat{p}}_{t,1},\ldots ,{\hat{p}}_{t,7}]$$

where ${\hat{p}}_{t,k}$ denotes the predicted probability that window t belongs to state $S_{k}$. To reduce the influence of single-window misclassification on event boundaries, causal temporal smoothing was first applied:

$${\overset{ˉ}{p}}_{t}=\frac{1}{K}\sum_{i=0}^{K-1}{\hat{p}}_{t-i}$$

where K=3, meaning that the current window and the previous two windows were used for smoothing. This corresponds to an approximately 30 s smoothing range and does not use future windows, making it applicable to both offline evaluation and real-time edge inference. The smoothed predicted label was defined as:

$${\overset{ˉ}{y}}_{t}=S_{arg\underset{k}{max}{\overset{ˉ}{p}}_{t,k}}$$

Derived state descriptors were also used to assist event extraction, including in-bed probability $P_{t}^{bed}$, stable sleep probability $P_{t}^{sleep}$, movement probability $P_{t}^{move}$, and nocturnal out-of-bed event or wake-up transition probability $P_{t}^{trans}$.

An in-bed transition was defined as a transition from an out-of-bed or unoccupied state to an in-bed state. If $P_{t}^{bed}\geq 0.5$ was satisfied for at least two consecutive windows and the preceding period was mainly labeled as $S_{0}$ or $S_{1}$, the start time of the first qualifying window was taken as the predicted in-bed transition time. Candidate in-bed events separated by less than 60 s were merged to avoid duplicate detections caused by short-term probability fluctuations.

A final out-of-bed transition was defined as a morning transition from an in-bed state to an out-of-bed or room-exit state, with no subsequent return to bed. If $P_{t}^{bed}<0.5$ was satisfied for at least two consecutive windows and the transition occurred after the wake-up transition or during the morning period, the start time of the first qualifying window was taken as the predicted final out-of-bed transition time. In offline event evaluation, this event was identified retrospectively. In real-time edge inference, the system only outputs the current out-of-bed transition status; whether it is the final out-of-bed transition requires confirmation using subsequent non-return-to-bed information.

A nocturnal out-of-bed event corresponded to the $S_{6}$ nocturnal out-of-bed state. A candidate event was generated if the smoothed $S_{6}$ probability satisfied

$${\overset{ˉ}{p}}_{t,6}\geq \theta_{6}$$

for at least two consecutive windows. The threshold was set to $\theta_{6}=0.4$ based on the validation set and then kept fixed for all event extraction analyses. The event onset was defined as the start time of the first qualifying $S_{6}$ window, and the event endpoint was defined as the last $S_{6}$ window before the state returned to the in-bed-related set $\left\{S_{2},S_{3},S_{4},S_{5}\right\}$. To avoid confusing final out-of-bed transitions with nocturnal out-of-bed events, a candidate event was required to have an in-bed or sleep-related state before it and a return-to-bed or in-bed state after it. Adjacent $S_{6}$ candidate segments separated by less than 60 s were merged into the same nocturnal out-of-bed event.

A wake-up transition corresponded to the $S_{7}$ wake-up transition state. Because wake-up transition is a gradual process rather than an instantaneous action, it was extracted as a continuous event interval. A candidate segment was generated if the smoothed $S_{7}$ probability satisfied

$${\overset{ˉ}{p}}_{t,7}\geq \theta_{7}$$

for at least three consecutive windows. The threshold was set to $\theta_{7}=0.4$ based on the validation set and then kept fixed for all event extraction analyses. The event onset was defined as the start time of the first qualifying window. The endpoint was defined as the final out-of-bed transition time or the time when the $S_{7}$ probability continuously decreased and the state shifted to out-of-bed event or unoccupied. To avoid misclassifying short nocturnal movements as wake-up transitions, the candidate segment was required to occur during the morning period and be followed by a final out-of-bed or room-exit state.

To evaluate whether event extraction was overly dependent on the validation-selected thresholds, an additional postprocessing sensitivity analysis was conducted. The trained SA-HMF model weights and window-level S0–S7 probability sequences were kept fixed. Only the event extraction parameters were varied. Specifically, θ6 for nocturnal out-of-bed detection and θ7 for wake-up transition detection were varied from 0.30 to 0.50 with a step of 0.05, and the causal smoothing length K was tested at 1, 3, and 5 windows. For each setting, event-level Precision, Recall, F1-score, and detection delay were recalculated using the same event-matching criteria. No threshold was re-selected during this sensitivity analysis; the default setting θ6 = 0.40, θ7 = 0.40, and K = 3 remained the operating point used for the main event extraction results.

For all event types, short-segment filtering and adjacent event merging were applied. Candidate in-bed, out-of-bed, or nocturnal out-of-bed segments shorter than 20 s were treated as short-term noise and removed. Wake-up transition candidates shorter than 60 s were removed. Adjacent predicted events of the same type separated by less than 60 s were merged. If multiple predicted events corresponded to the same ground-truth event, only the closest prediction was counted as a true positive, and the remaining predictions were counted as false positives.

Detection delay was calculated based on event onset time. Let $T_{i}^{gt}$ denote the manually annotated event onset time and $T_{i}^{pred}$ denote the predicted event onset time. The detection delay was defined as:

$$D_{i}=T_{i}^{pred}-T_{i}^{gt}$$

For events with clear onset times, including in-bed transition, final out-of-bed transition, and nocturnal out-of-bed states, $D_{i}$ represents the time difference between the true event onset and model detection. For gradual wake-up transitions, onset error was also computed using the start times of the predicted and annotated transition intervals, and event matching additionally considered temporal overlap. Event-level Precision, Recall, and F1-score were calculated from matched predicted and annotated events. Point-like transition events were treated as matched when the onset-time difference was within 30 s, whereas wake-up transition intervals were treated as matched when the predicted and annotated intervals overlapped by at least 50%.

Through this postprocessing pipeline, window-level state probability sequences were converted into evaluable event sequences.

#### 2.6.4. Edge Implementation

To evaluate the deployability of SA-HMF in a practical bedroom sensing system, the trained model was deployed on a local edge gateway for inference testing. The edge platform used a Raspberry Pi 4B-class gateway (Raspberry Pi Ltd, Cambridge, UK) or an equivalent low-power edge device [40]. The gateway received data from all sensing nodes, maintained a sliding window buffer for the most recent 30 s, constructed window-level features, executed SA-HMF inference, and output the S0–S7 sleep-related bedroom state probabilities for the current window.

The edge inference update interval was the same as the sliding stride, namely, one state update every 10 s. For each inference, the edge device read the most recent 30 s of multimodal data from the buffer, performed temporal alignment, read missingness markers, constructed window-level features, standardized continuous features using training set statistics, and then fed the features into the SA-HMF main classifier. The model output the current state probability vector:

$${\hat{p}}_{t}=[{\hat{p}}_{t,0},{\hat{p}}_{t,1},\ldots ,{\hat{p}}_{t,7}]$$

The gateway further computed derived state descriptors, such as in-bed probability, stable sleep probability, movement probability, and nocturnal out-of-bed or wake-up transition probability. The edge output consisted of state recognition results and state descriptors, rather than direct ventilation, lighting, thermal regulation, or energy optimization commands. Control-related inference was performed separately in the downstream application layer.

To distinguish algorithmic performance from engineering overhead, an unoptimized SA-HMF implementation was additionally recorded as a deployment overhead baseline. This implementation used the same S0–S7 main classification structure and the same trained weights as the proposed SA-HMF but did not use the lightweight inference configuration or runtime optimizations of the final edge deployment version. Therefore, it was used only to compare model size, inference latency, and memory usage and was not treated as an additional state recognition algorithm baseline.

During edge deployment, model file size, single-window inference latency, memory usage, window update interval, packet missingness, and the proportion of low-confidence outputs were recorded to evaluate feasibility on the local low-power bedroom gateway. Inference latency was measured per input window and compared with the 10 s state update interval. If single-window inference latency was substantially lower than 10 s, the model was considered capable of real-time state updating.

For missing data, the edge device did not treat missing values as normal observations. Instead, missingness markers were retained. Short missing segments in low-frequency IEQ variables could be filled using neighboring valid values or window-level interpolation. For key modalities, such as bed-load sensing, bed-mounted accelerometry, and contextual events, missingness markers were retained and passed to the gated fusion module, allowing the model to reduce the influence of unavailable modalities. If a window contained severe missingness in key modalities, it was marked as a low-confidence state output and analyzed separately in robustness evaluation.

Edge inference was mainly performed locally on the gateway and did not require uploading raw bedroom sensing data to the cloud. The system stored only necessary anonymized window-level features, state probabilities, event outputs, and system operation logs. This implementation helps reduce privacy risks in nocturnal bedroom scenarios and improves the deployment acceptability of furniture-embedded multimodal sensing systems in real homes. The corresponding results for these validation protocols are reported in Section 3.4.

### 2.7. Exploratory Downstream Application Analyses

Predicted SA-HMF state probabilities and derived sleep-aware descriptors were further examined in exploratory downstream analyses to assess whether fine-grained S0–S7 state information provided additional contextual value beyond conventional room-level occupancy. These analyses included short-term microenvironment prediction, rule-defined application proxies, and retrospective offline replay. They were not used to develop or validate a prospective closed-loop controller.

#### 2.7.1. Microenvironment Prediction

CO_2_ concentration and air temperature were predicted 10 min ahead using environmental, contextual, temporal, and historical actuator state information. Three settings were compared under the same evaluation protocol: (1) no sleep-aware state information, (2) room-level occupancy information, and (3) SA-HMF state probabilities and derived descriptors. Performance was evaluated using MAE and RMSE. This analysis was used only to quantify the additional contextual information provided by the recognized states.

#### 2.7.2. Rule-Defined Application Proxies and Retrospective Replay

SA-HMF outputs were additionally evaluated for four rule-defined proxy tasks: ventilation demand, thermal regulation demand, lighting demand, and actuator disturbance risk (Table 5). These labels were derived from environmental, contextual, state, and actuator information and should not be interpreted as direct measurements of occupant demand, physiological disturbance, or optimal control actions.

A limited retrospective replay was also conducted using recorded sensing, actuator, and energy logs under predefined rules. No real-time control commands were issued; therefore, the replay was used only to illustrate potential downstream relevance and did not constitute validation of a control algorithm or prospective closed-loop strategy.

### 2.8. Experimental Evaluation Design

To systematically evaluate the proposed furniture-embedded multimodal sensing framework and the SA-HMF model, five groups of experiments were designed. First, SA-HMF was compared with rule-based methods, single-modality models, room-level sensing models, conventional machine learning models, temporal models, and structural variants of SA-HMF. Second, window-level multiclass metrics and class-wise metrics were used to evaluate S0–S7 state recognition performance. Third, event-level metrics were used to evaluate key events, including in-bed transition, final out-of-bed transition, nocturnal out-of-bed event, and wake-up transition. Fourth, missing data, modality dropout, sensor degradation, and edge inference experiments were used to assess robustness and deployment feasibility. Fifth, microenvironment prediction, control-demand inference, and offline replay metrics were used to evaluate the application value of the recognized states.

Because nocturnal bedroom states are naturally imbalanced, $S_{4}$ stable sleep is the majority class, whereas $S_{6}$ nocturnal out-of-bed event and $S_{7}$ wake-up transition are low-frequency but control-relevant states. Therefore, Accuracy was not used as the only evaluation metric; Macro-F1, Balanced Accuracy, class-wise F1-score, event-level F1, and detection delay were emphasized.

#### 2.8.1. Baseline Methods and Ablation Settings

All baseline and ablation experiments used the same window length, sliding stride, train/validation/test split, feature construction procedure, and evaluation metrics. The baseline methods were used to evaluate whether the proposed method outperformed common alternatives, whereas the ablation settings were used to analyze the contributions of bed-interaction sensing, multimodal inputs, hierarchical structure, and attention-gated fusion. The baseline methods and controlled ablation settings are summarized in Table 6.

All baseline and ablation models used the same window definition, data split, preprocessing procedure, and evaluation metrics. The modality ablations assessed the contribution and complementarity of the sensing inputs, whereas the structural ablations isolated the bed-conditioned hierarchy and attention-gated fusion. The structural variants were controlled comparisons rather than independently redesigned models. The full, non-hierarchical, and no-attention/gate models contained 672,418, 654,730, and 660,982 trainable parameters, respectively. The full and non-hierarchical models differed by only 2.63% in parameter count, reducing model capacity as an alternative explanation for their performance difference.

Two additional ablations were conducted only in the downstream application layer. One removed the SA-HMF state descriptors from environmental prediction and control-demand inference; the other removed the actuator disturbance penalty from offline replay. These settings were not treated as ablations of the S0–S7 recognition model.

#### 2.8.2. State Recognition, Event Detection, and Generalization Metrics

For the S0–S7 window-level multiclass recognition task, overall performance was evaluated using Accuracy, Macro-F1, Weighted-F1, and Balanced Accuracy. Class-wise Precision, Recall, and F1-score were also reported. Precision, Recall, and F1-score were calculated using standard TP, FP, and FN definitions. Macro-F1 was the arithmetic mean of class-wise F1-scores, Weighted-F1 was weighted by class sample size, and Balanced Accuracy was the mean of class-wise Recall values. Because of class imbalance, Macro-F1 and Balanced Accuracy were prioritized in overall model comparison, while class-wise analysis focused on $S_{6}$, $S_{7}$, and easily confused state pairs such as $S_{4}/S_{5}$, $S_{1}/S_{6}$, and $S_{2}/S_{7}$.

In addition to window-level classification, key sleep-related events were evaluated at the event level. The evaluated events included in-bed transition, final out-of-bed transition, nocturnal out-of-bed event, and wake-up transition. Event-level evaluation used Precision, Recall, F1-score, detection delay, and onset error. For events with clear onset times, including in-bed transition, final out-of-bed transition, and nocturnal out-of-bed event, a predicted event was considered matched if the difference between the predicted onset time and the manually annotated onset time was no more than 30 s. For gradual wake-up transition events, a predicted interval was considered matched if it overlapped with the annotated interval by at least 50%. If multiple predicted events corresponded to the same ground-truth event, only the temporally closest prediction was counted as a true positive, and the remaining predictions were counted as false positives.

The validation protocols were interpreted according to their inferential scope. Night-independent splitting evaluated cross-night stability within the same participants and bedroom. Subject-independent splitting evaluated transfer to unseen participants within the tested bedroom and sensing configuration. LOSO characterized participant-level variability within the same setting.

Because overlapping windows were correlated, statistical inference was performed at the participant level. Full SA-HMF and the two structural variants were evaluated using the same 20 LOSO folds. Macro-F1 was calculated separately for each held-out participant. Means, standard deviations, medians, and 95% confidence intervals were reported, with confidence intervals and paired differences estimated using 10,000 participant-level bootstrap resamples.

Full SA-HMF was compared with each structural variant using two-sided Wilcoxon signed-rank tests. Holm correction was applied to the two comparisons. No significance test treated overlapping windows as independent observations.

#### 2.8.3. Robustness, Deployment, and Application Layer Metrics

Robustness evaluation assessed model stability under practical sensing degradation, including short-term missing data, wireless packet loss, node failure, and sensor disturbance. Three degradation conditions were tested: random missing, modality dropout, and observed sensor noise/drift. In the random missing setting, 10%, 20%, and 30% of the input data were randomly removed. In the modality dropout setting, the bed node, IEQ node, or context node was removed separately. In the sensor noise/drift setting, noisy, fluctuating, or drift-affected continuous sensor segments observed in the experimental records were used for evaluation. Random missing experiments were repeated with five random seeds, and average performance was reported. Robustness was evaluated mainly using Accuracy, Macro-F1, $S_{6}$ F1, and $S_{4}$ F1 to reflect both low-frequency key event states and the majority stable-sleep state.

In addition to sensing degradation robustness, event extraction robustness was evaluated by varying the postprocessing probability thresholds and causal smoothing length used for nocturnal out-of-bed event and wake-up transition detection. The trained model outputs were fixed, and only θ6, θ7, and K were changed to quantify the sensitivity of event-level F1-scores and detection delays.

Deployment evaluation examined whether SA-HMF could run on a local low-power bedroom gateway. The metrics included model file size, single-window inference latency, memory usage, window update interval, and the ratio of inference latency to the 10 s sliding stride. If single-window inference latency was substantially lower than 10 s, the model was considered suitable for real-time state updating. Sensing network quality was described using modality missing rates, communication packet loss rate, and time synchronization error.

Application layer evaluation examined whether SA-HMF state outputs supported downstream environmental prediction and control-demand inference. The microenvironment prediction task evaluated 10 min ahead CO_2_ concentration and air temperature prediction using MAE and RMSE and compared three input settings: without sleep-aware state, with room-level occupancy, and with SA-HMF state output. The control-demand inference task evaluated ventilation demand, thermal regulation demand, lighting demand, and actuator disturbance risk using Accuracy and Macro-F1. Offline replay evaluation compared normalized measured energy use, CO_2_ exceedance time, temperature discomfort time, and actuator actions during stable sleep. The complete definitions of robustness, edge deployment, and application layer metrics are summarized in Supplementary Table S7.

## 3. Results

### 3.1. Dataset Characteristics and Signal Quality

Twenty healthy adults participated in the study, including 10 males and 10 females. Their ages ranged from 19 to 53 years, with a mean age of 31.8 ± 8.6 years. A total of 140 participant-nights were planned, and 137 nights were successfully recorded. After night-level and long-segment-level quality control, 127 valid participant-nights were retained, corresponding to 92.7% of the successfully recorded nights. The total raw recording duration was 1094.8 h, including invalid nights or segments that were later excluded.

The synchronized recording segments retained after night-level and long-segment-level quality control were segmented using 30 s windows with a 10 s sliding stride, generating 365,921 candidate windows. Based on the 10 s stride, these windows corresponded to a candidate window stride-based equivalent coverage of 1016.4 h. After window-level quality screening and label verification, 23,237 windows with severe missingness, abnormal values, or uncertain labels were excluded, accounting for approximately 6.35% of the candidate windows. Finally, 342,684 valid labeled windows were retained for model training, validation, and testing, accounting for 93.65% of the candidate windows. Based on the 10 s stride, the valid window stride-based equivalent duration was 951.9 h. These stride-based values describe the scale of overlapping window samples and should not be interpreted as non-overlapping real recording duration. The complete dataset construction statistics, including participant information, recorded nights, window-level screening, and event counts, are summarized in Supplementary Table S8.

Overall, the sensing modalities showed high data coverage. Door-contact and PIR sensors had the lowest missing rates, at 0.9% and 1.4%, respectively. The missing rates of air temperature, relative humidity, and illuminance were 1.9%, 2.0%, and 2.7%, respectively. Bed-load sensing and bed-mounted accelerometry had missing rates of 2.2% and 3.1%, respectively. The CO_2_ module showed a relatively higher missing rate of 4.4%, mainly due to sensor warm-up and communication delay. The average packet-loss rate was 1.84%, and the mean time synchronization error was 0.46 ± 0.31 s, which was substantially smaller than the 30 s window length and 10 s sliding stride. Therefore, its influence on window-level state recognition was limited. The full summary of data coverage, missing rates, and synchronization quality is provided in Supplementary Table S9.

**Figure 5:**
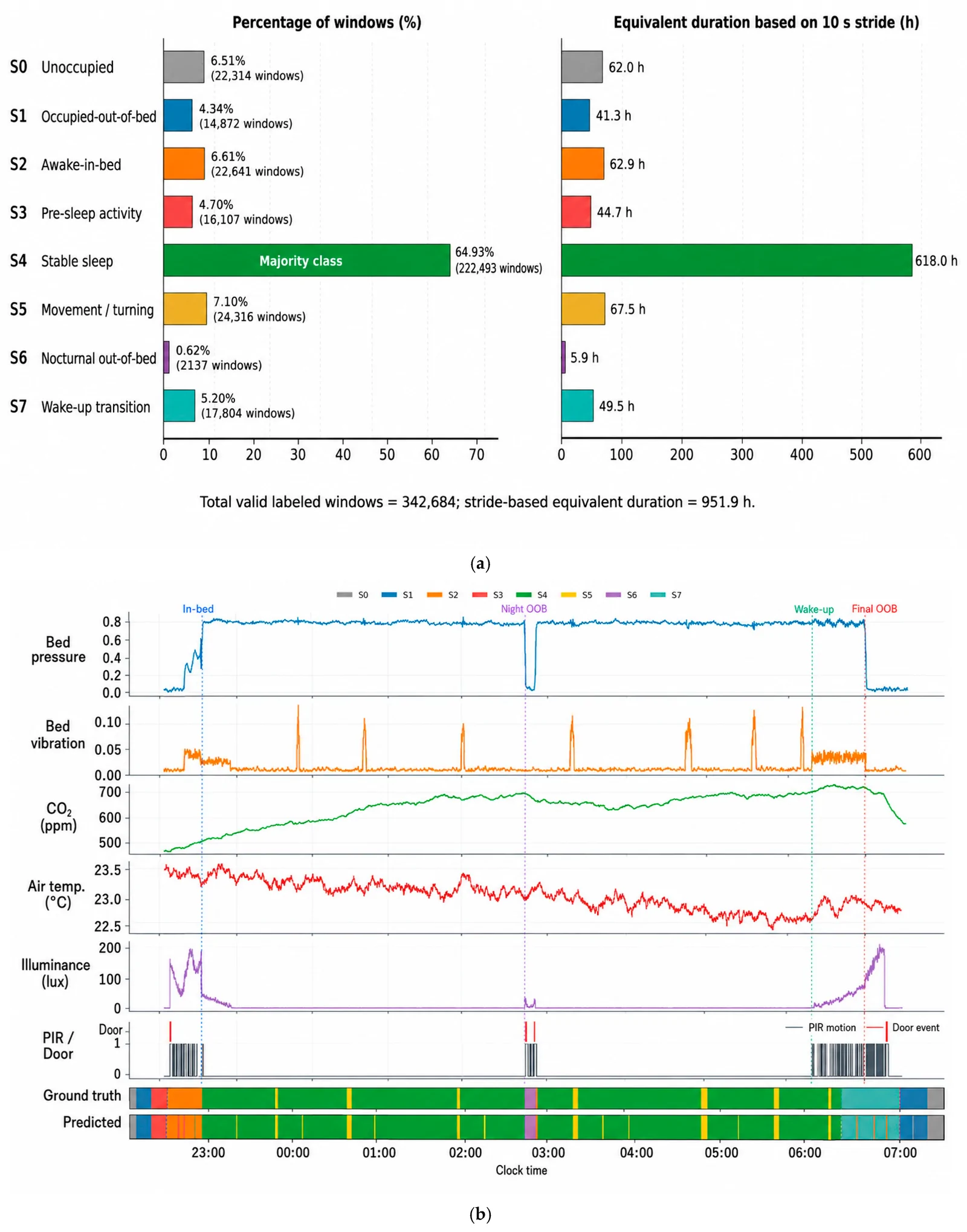
Dataset state distribution and representative overnight multimodal sensing sequence. (**a**) Distribution of S0–S7 control-oriented sleep-related bedroom states. (**b**) Representative multimodal time series and S0–S7 state labels over one night.

Figure 5 shows the window-level distribution of the S0–S7 state labels. The dataset exhibited clear natural class imbalance. $S_{4}$ stable sleep was the majority class, with 222,493 windows, accounting for 64.93% of all valid labeled windows. In contrast, $S_{6}$ nocturnal out-of-bed event was the lowest frequency key state, with 2137 windows, accounting for 0.62%. This distribution is consistent with real nocturnal bedroom behavior, in which stable sleep occupies most of the night, whereas nocturnal out-of-bed events are infrequent but control-relevant. Therefore, subsequent model evaluation reports not only Accuracy but also Macro-F1, Balanced Accuracy, and event-level F1 to reduce the dominance of majority class performance.

### 3.2. State Recognition and Event Detection Performance

Table 7 reports point estimates under the primary subject-independent split. Full SA-HMF achieved 0.925 Accuracy, 0.874 Macro-F1, 0.927 Weighted-F1, and 0.864 Balanced Accuracy, exceeding the rule-based, single-source, early fusion, GRU, and non-hierarchical comparisons within the tested setting.

Removing the bed-centered hierarchy reduced Macro-F1 from 0.874 to 0.856, whereas removing attention/gating reduced it to 0.861. These values are point estimates from the predefined subject-independent split. Participant-level consistency and statistical uncertainty were evaluated separately using the same 20 LOSO folds, as reported in Supplementary Table S14.

The bed-only model clearly outperformed the room-level sensing model, showing that bed-load and accelerometry-derived bed-motion features were critical for identifying in-bed, out-of-bed, stable sleep-related, and movement/turning states. However, bed-interaction sensing alone was not sufficient for all S0–S7 states, especially for distinguishing prolonged unoccupied and occupied-out-of-bed states. Therefore, the results support the complementarity of bed-interaction, room-context, IEQ, and time features for control-oriented bedroom state recognition.

In addition to window-level classification, retrospective event extraction was performed across all 127 valid participant-nights to examine whether the smoothed SA-HMF state probability sequences could recover key sleep-related events from complete nocturnal sequences. The event-level analysis included in-bed transition, final out-of-bed transition, nocturnal out-of-bed event, and wake-up transition. Because each valid night contributed one initial in-bed transition and one final out-of-bed transition, these two event types each contained 127 events. Temporary nocturnal out-of-bed events followed by return to bed were not counted as final out-of-bed transitions but were separately counted as nocturnal out-of-bed events, with 41 events in total. The event-level results in Table 8 should be interpreted as retrospective sequence-level analysis across the full dataset, whereas subject-independent generalization is mainly reflected by the window-level state recognition results in Table 7.

In-bed transition and final out-of-bed transition achieved the best event detection performance, with F1-scores of 0.950 and 0.935, and mean detection delays of 5.8 ± 3.1 s and 7.2 ± 4.0 s, respectively. This indicates that bed-load signals responded rapidly to getting-into-bed and final leaving-bed actions. For the 41 nocturnal out-of-bed events, event-level Precision, Recall, and F1-score were 0.864, 0.829, and 0.846, respectively, with a mean detection delay of 11.6 ± 7.4 s. Although $S_{6}$ nocturnal out-of-bed event was a low-frequency state, SA-HMF effectively detected nocturnal leaving-bed behavior by combining bed-load reduction, PIR or door-contact events, and nighttime context. Wake-up transition included 112 events and achieved an F1-score of 0.860, with a mean detection delay of 26.4 ± 18.2 s. Because wake-up transition is a gradual process rather than an instantaneous action, its longer detection delay is reasonable.

To test the robustness of the validation-selected event extraction parameters, θ6, θ7, and K were varied while keeping the trained SA-HMF outputs unchanged. As shown in Supplementary Table S12, nocturnal out-of-bed F1 remained within 0.813–0.846 when θ6 ranged from 0.30 to 0.50, and wake-up transition F1 remained within 0.829–0.860 when θ7 ranged from 0.30 to 0.50. Changing K from 1 to 5 also caused only limited F1 variation, although a larger K increased detection delay. These results suggest that the default setting θ6 = 0.40, θ7 = 0.40, and K = 3 provided a balanced operating point rather than a narrowly tuned threshold combination.

**Figure 6:**
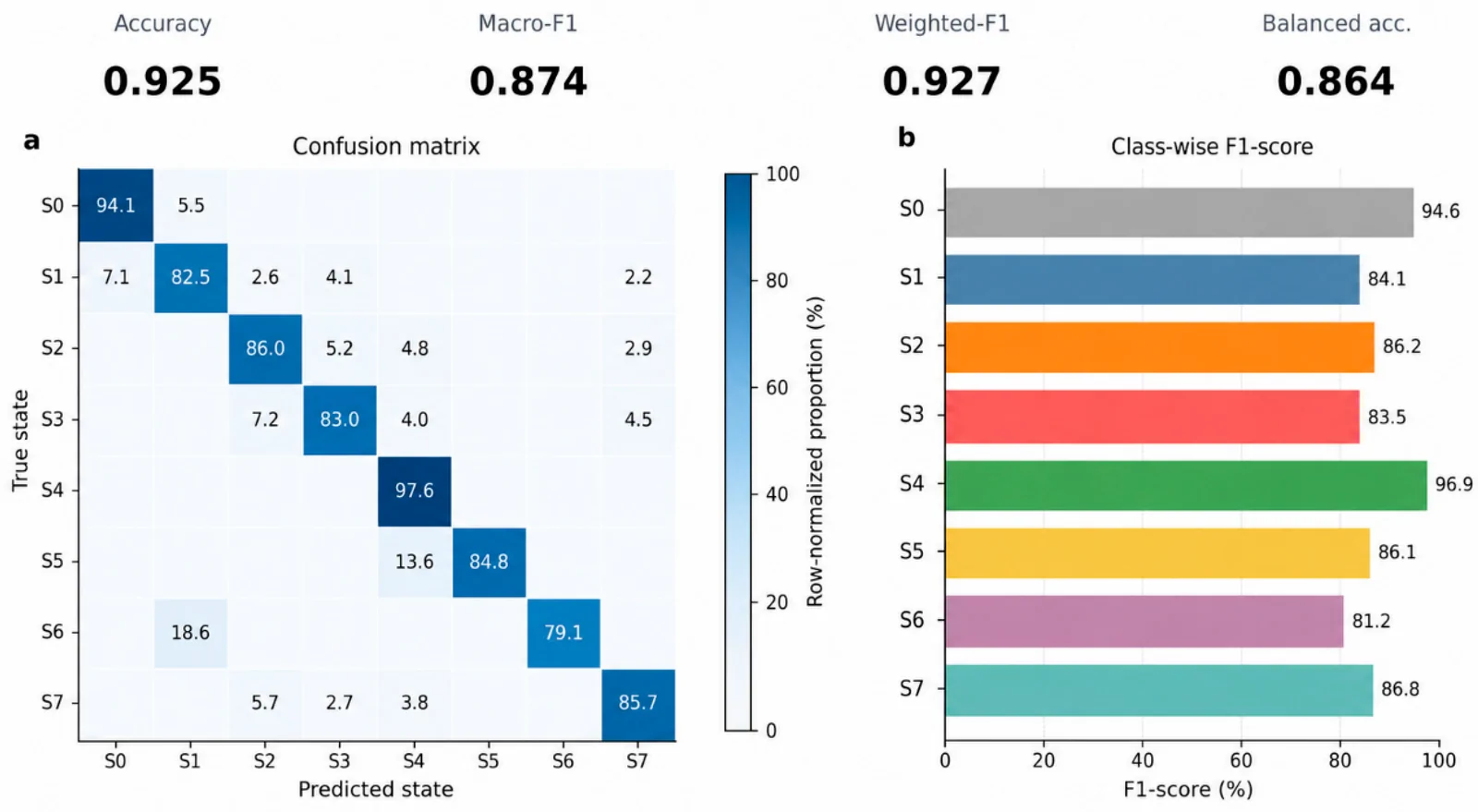
Subject-independent state recognition performance of SA-HMF. The top summary reports Accuracy, Macro-F1, Weighted-F1 and Balanced Accuracy under the subject-independent split. Panel (**a**) shows the row-normalized confusion matrix for S0–S7 state recognition, and panel (**b**) shows the class-wise F1-score.

Figure 6 further shows the class-wise recognition performance and confusion structure of the proposed SA-HMF. The model achieved the best performance for $S_{4}$ stable sleep, with an F1-score of 0.969, which is consistent with its large sample size and stable bed-interaction patterns. $S_{0}$ unoccupied achieved an F1-score of 0.946, indicating that bed-load, PIR, and door-contact information can reliably distinguish the unoccupied state. $S_{5}$ movement/turning achieved an F1-score of 0.861, suggesting that accelerometry-derived bed-motion features effectively captured body movement and turning.

For transition states and low-frequency events, SA-HMF still maintained good recognition performance. $S_{6}$ nocturnal out-of-bed event achieved an F1-score of 0.812. Although this value was lower than those of the majority states, $S_{6}$ accounted for only 0.62% of all windows, indicating that the model could still capture nocturnal out-of-bed events effectively. $S_{7}$ wake-up transition achieved an F1-score of 0.868, showing that the model could identify gradual morning wake-up processes by combining morning time context, increased bed-motion, and illuminance changes. $S_{2}$ awake-in-bed and $S_{3}$ pre-sleep activity states achieved F1-scores of 0.862 and 0.835, respectively. These two states were more easily confused because both can occur during awake periods after getting into bed and may show similar bed-load and illuminance patterns.

The row-normalized confusion matrix in Figure 6 shows that SA-HMF errors were concentrated in five main confusion patterns rather than being randomly distributed across all classes. First, $S_{0}$ unoccupied and $S_{1}$ occupied-out-of-bed states showed slight bidirectional confusion because both states had low bed load and required PIR and door-contact context for further distinction. Second, $S_{2}$ awake-in-bed and $S_{3}$ pre-sleep activity states were easily confused because both may occur during awake periods after getting into bed and share similar bed-load and illuminance characteristics. Third, some $S_{5}$ movement/turning windows were classified as $S_{4}$ stable sleep, suggesting that weak or short-duration body movements may become close to stable sleep-related states after temporal smoothing. Fourth, $S_{6}$ nocturnal out-of-bed event was mainly confused with $S_{1}$ occupied-out-of-bed state because both states showed low bed load; distinguishing them requires nighttime context and subsequent return-to-bed information. Fifth, $S_{7}$ wake-up transition showed some confusion with the $S_{2}$ awake-in-bed state, reflecting that the morning wake-up transition is a gradual process rather than an instantaneous state change.

**Figure 7:**
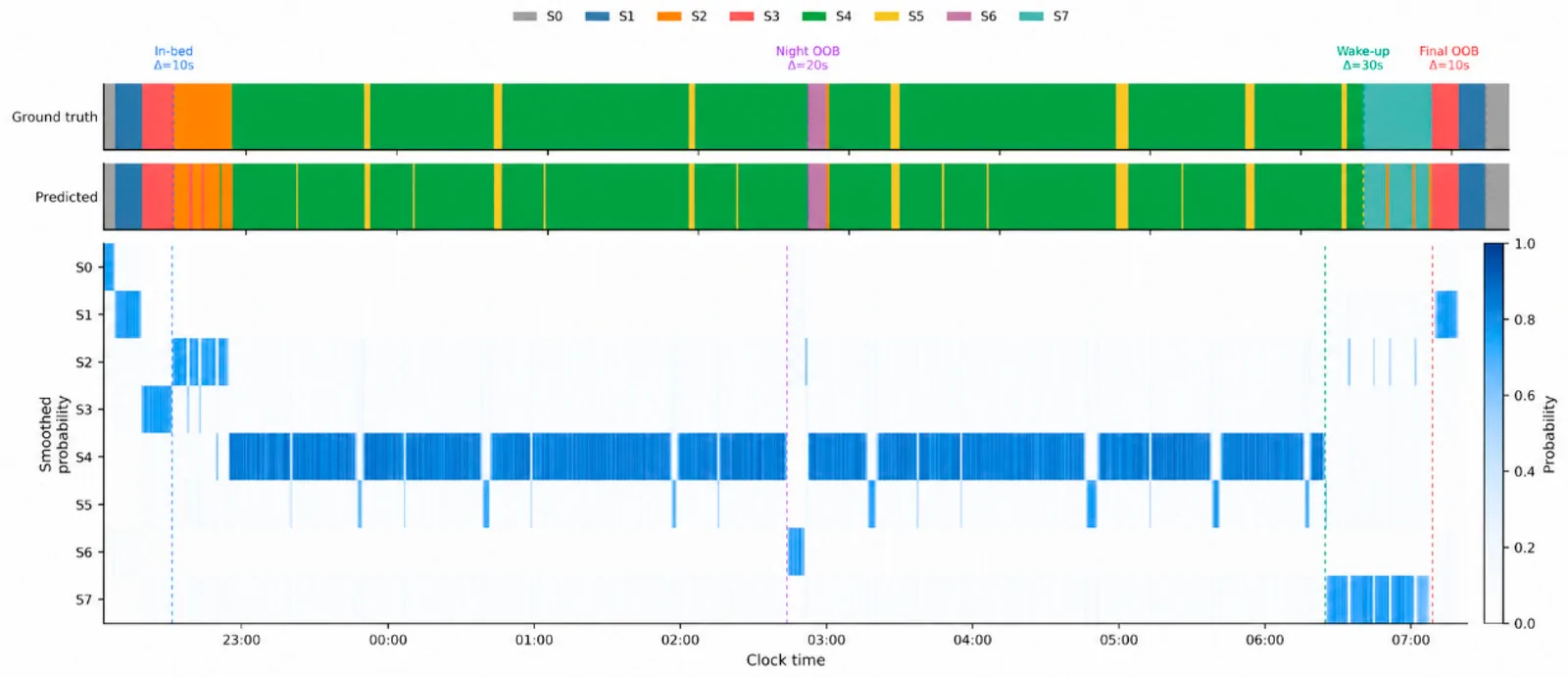
Representative night state trajectories and smoothed state probabilities. The top two strips show the ground-truth and predicted S0–S7 state timelines, while the heatmap shows the smoothed probability of each state over the same night. Vertical dashed lines indicate the four evaluated transition events, including in-bed transition, nocturnal out-of-bed episode, wake-up transition and final out-of-bed transition. Detection delays are shown above the corresponding event markers.

Figure 7 shows the continuous state recognition results for a representative night. Overall, SA-HMF tracked the major state changes well. The ground-truth and predicted state timelines were generally consistent across the in-bed transition, stable sleep, short nocturnal out-of-bed episode, morning wake-up transition, and final out-of-bed transition. The smoothed probability heatmap further shows that $S_{4}$ stable sleep dominated the main nocturnal sleep period, $S_{6}$ nocturnal out-of-bed event increased briefly during the nocturnal out-of-bed episode, and $S_{7}$ wake-up transition gradually increased during the morning wake-up period. Although small temporal boundary deviations occurred during short nocturnal out-of-bed and morning wake-up transition periods, the predicted state types were generally consistent with the ground truth.

Together, Table 7 and Figure 6 and Figure 7 show that SA-HMF recognized the operational bedroom states and supported event extraction within the tested bedroom and sensing configuration.

### 3.3. Modality Contribution and Structural Ablation

To evaluate the contributions of different modalities and model structures, modality ablation and structural ablation experiments were conducted. As shown in Table 9, the bed-only model achieved a Macro-F1 of 0.694, outperforming IEQ-only (0.438) and context-only (0.582). This indicates that bed-load and accelerometry-derived bed-motion features are the core modalities for recognizing sleep-related bedroom states. The IEQ-only setting showed the lowest performance, suggesting that CO_2_ concentration, air temperature, relative humidity, and illuminance alone are insufficient to distinguish bed-interaction states such as in-bed, stable sleep-related, and movement/turning states.

The modality combination results further show the benefit of multimodal inputs. Bed + IEQ achieved a Macro-F1 of 0.753, improving upon bed-only. Bed + context + time achieved a Macro-F1 of 0.826, indicating that PIR, door-contact events, and time features are important for distinguishing states such as occupied-out-of-bed, nocturnal out-of-bed, and wake-up transition. Bed + IEQ + context + time achieved a Macro-F1 of 0.846, confirming that multimodal inputs outperform single-modality or partial-modality configurations.

The structural ablation results show that both the hierarchical structure and attention-gated fusion contributed to SA-HMF performance. SA-HMF without hierarchy achieved a Macro-F1 of 0.856, and SA-HMF without attention/gate achieved a Macro-F1 of 0.861, whereas the full proposed SA-HMF reached 0.874. Although the structural gains were smaller than the gains from adding key modalities, they were consistent under the subject-independent split, suggesting that hierarchical recognition and adaptive multimodal fusion improve cross-subject state recognition performance.

Bed interaction remained the primary information source, while context and time features supported out-of-bed and transition state recognition. Under IEQ node and context node dropout, full SA-HMF retained Macro-F1 values of 0.857 and 0.843, compared with 0.839 and 0.817 without attention/gating. These results are consistent with the intended roles of the architecture: the hierarchy supports conditional state interpretation, whereas adaptive gating improves robustness to degraded modalities. Detailed results are reported in Supplementary Table S13.

### 3.4. Deployment-Oriented Robustness, Generalization, and Edge Performance

Real bedroom deployment inevitably involves wireless packet loss, short-term missing data, temporary node failures, and non-missing sensor degradation. Therefore, the robustness of SA-HMF was evaluated under three degradation conditions: random missing, modality dropout, and observed sensor noise/drift. Random missing and modality dropout were generated by offline masking of valid experimental data, whereas sensor noise/drift was evaluated using noisy, fluctuating, or drift-affected segments observed in the experimental records.

Under nominal sensing conditions, SA-HMF achieved an Accuracy of 0.925 and a Macro-F1 of 0.874. When the random missing rate increased to 10%, 20%, and 30%, Macro-F1 decreased to 0.858, 0.832, and 0.801, respectively. Although performance declined as missingness increased, the model still maintained a Macro-F1 of 0.801 under 30% random missing, indicating tolerance to incomplete sensing inputs. Compared with $S_{4}$ stable sleep, $S_{6}$ nocturnal out-of-bed event was more sensitive to missing data, which is consistent with its low-frequency nature and stronger dependence on bed-interaction and contextual evidence.

**Figure 8:**
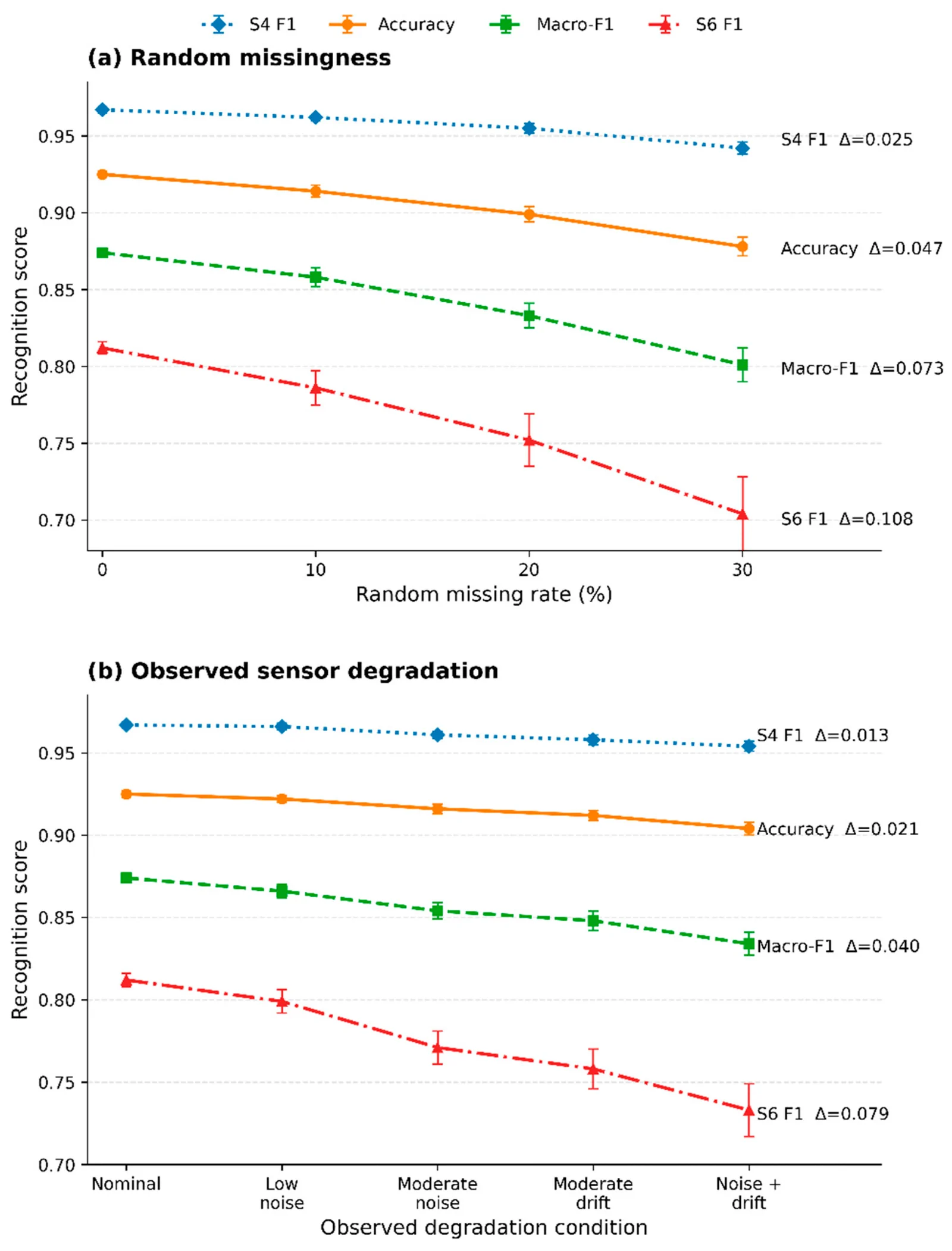
Robustness under missing data and observed sensor degradation conditions. (**a**) Performance degradation under random missing conditions. (**b**) Performance under observed sensor noise and drift conditions. Random missing rate was evaluated through offline masking, whereas sensor noise/drift was evaluated using noisy or drift-affected segments observed in the experimental recordings. Error bars indicate ± SD.

Under observed sensor noise and drift, low-level sensor noise caused only a slight performance decrease, with Accuracy and Macro-F1 decreasing from 0.925 and 0.874 to 0.921 ± 0.002 and 0.866 ± 0.002, respectively. Under combined noise and drift, Accuracy and Macro-F1 decreased to 0.904 ± 0.003 and 0.834 ± 0.005. The decline was more pronounced for $S_{6}$ nocturnal out-of-bed event, whose F1-score decreased from 0.812 to 0.733 ± 0.010. In contrast, $S_{4}$ stable sleep remained relatively stable, with its F1-score decreasing only from 0.969 to 0.956 ± 0.003. These results indicate that low-frequency transition-related states are more vulnerable to sensing degradation than stable sleep-related states. The effects of random missingness and observed sensor degradation on recognition performance are summarized in Figure 8.

Modality dropout confirmed that the bed-interaction node was the most critical sensing component. Removing this node reduced Macro-F1 to 0.587 and S6 F1 to 0.421. By comparison, the effects of IEQ node and context node dropout were smaller, confirming that bed interaction provides the primary state evidence, while room context supplies complementary occupancy and transition cues. Detailed node dropout results are reported in Supplementary Table S13.

Performance varied across validation protocols. The random window split achieved an Accuracy of 0.949, a Macro-F1 of 0.911, and a Weighted-F1 of 0.951, but it was retained only as a potentially optimistic reference because overlapping neighboring windows could occur in both training and test sets. The night-independent split achieved an Accuracy of 0.931, a Macro-F1 of 0.884, and a Weighted-F1 of 0.932, indicating cross-night stability within the same participants and bedroom.

Under the primary subject-independent split, SA-HMF achieved an Accuracy of 0.925, a Macro-F1 of 0.874, and a Weighted-F1 of 0.927, supporting transfer to unseen participants within the tested bedroom and sensing configuration. LOSO evaluation yielded an Accuracy of 0.914 ± 0.029, a Macro-F1 of 0.852 ± 0.043, and a Weighted-F1 of 0.916 ± 0.030. The median LOSO Macro-F1 was 0.858, with a participant-level 95% confidence interval of 0.833–0.870. Compared with the non-hierarchical model, the paired Macro-F1 improvement was 0.017 (95% CI: 0.006–0.028), and the full model performed better for 16 of 20 participants ($p_{Holm}=0.024$). Compared with the model without attention/gating, the improvement was 0.010 (95% CI: −0.001–0.020), with better performance for 14 of 20 participants; this difference was not statistically significant ($p_{Holm}=0.082$).

**Figure 9:**
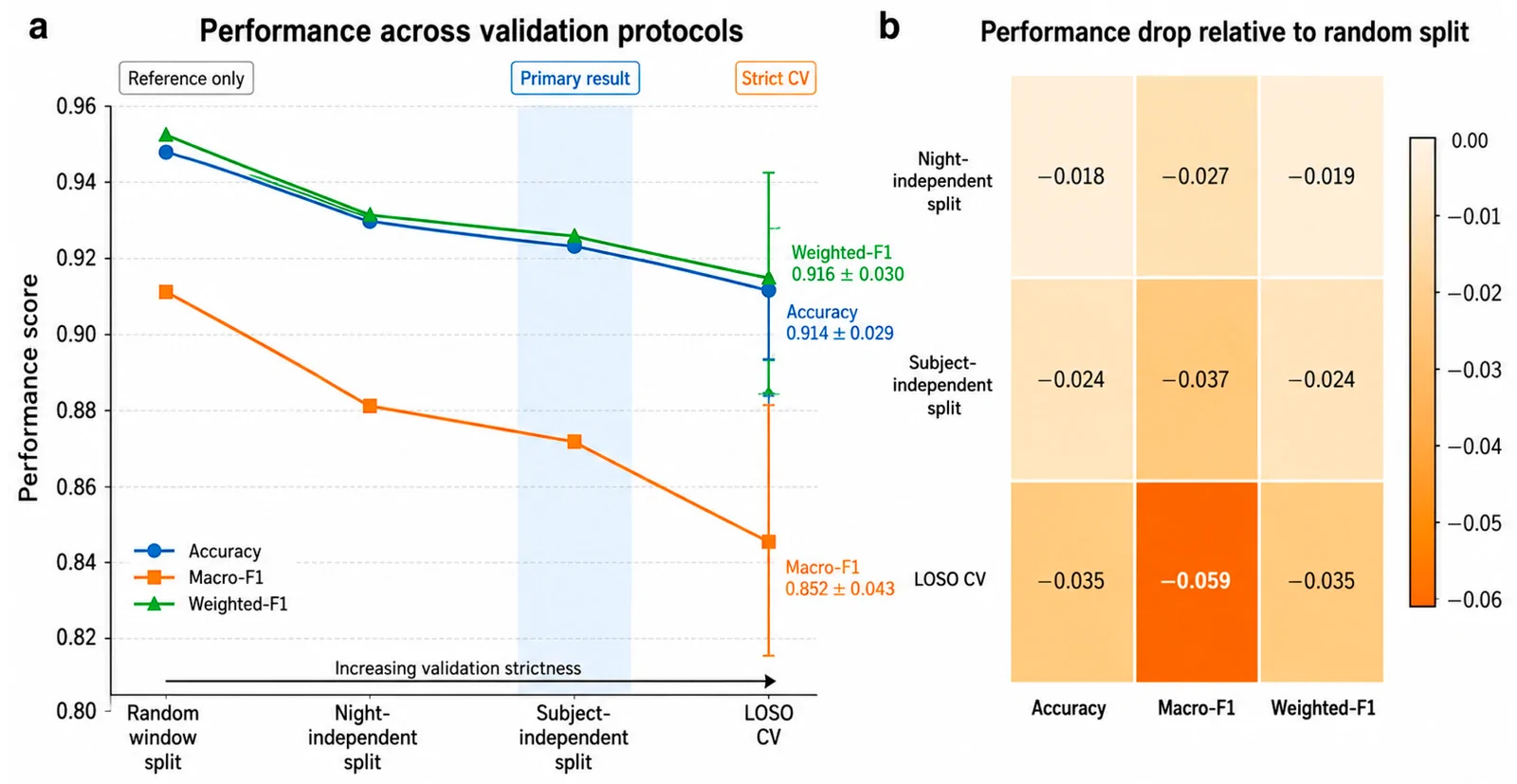
Generalization performance under different validation protocols. (**a**) Accuracy, Macro-F1 and Weighted-F1 across random window split, night-independent split, subject-independent split and leave-one-subject-out cross-validation (LOSO CV). Random window split is shown as a reference setting, while subject-independent split is highlighted as the primary evaluation protocol. Error bars are shown only for LOSO CV, where the results are reported as mean ± SD. (**b**) Absolute performance drop relative to the random window split. The larger drop under stricter protocols indicates that random window split provides a more optimistic estimate of performance. Subject-independent and LOSO evaluations better reflect transfer to unseen participants and participant-level variability within the tested setting; they do not represent cross-bedroom validation.

Using non-overlapping 30 s windows reduced the dataset from 342,684 to 114,126 instances. Full SA-HMF retained an Accuracy of 0.918, a Macro-F1 of 0.865, and a Balanced Accuracy of 0.856, whereas the non-hierarchical model achieved 0.906, 0.848, and 0.846, respectively. The full model lost only 0.009 Macro-F1, and its advantage over the non-hierarchical model was preserved. Thus, the primary performance pattern was not solely attributable to repeated sampling of adjacent windows. This sensitivity analysis does not establish cross-bedroom external validity. The performance and interpretation of the different validation protocols are summarized in Table 10. The corresponding generalization performance across the different validation protocols is further illustrated in Figure 9.

Together, the robustness and validation results show that SA-HMF retained useful performance under incomplete sensing, observed degradation, held-out nights, and unseen participants within the tested setting. Bed node loss produced the largest degradation. The non-overlapping analysis further showed that adjacent window overlap did not solely explain the primary performance pattern.

Edge implementation was further evaluated to assess whether SA-HMF could run on a local low-power bedroom gateway. Table 11 summarizes the deployment performance of different models. LightGBM had the lowest computational overhead, with a model size of 4.8 MB, inference latency of 6.4 ± 1.2 ms, and memory usage of 74 MB. The GRU temporal model had a model size of 1.9 MB, inference latency of 18.7 ± 3.6 ms, and memory usage of 132 MB. The unoptimized SA-HMF implementation had a model size of 3.4 MB, inference latency of 21.8 ± 4.1 ms, and memory usage of 146 MB. This implementation used the same S0–S7 classification structure and trained weights as the proposed model, but it did not include the lightweight inference configuration or runtime optimization used in the final edge version; therefore, it served only as a deployment overhead comparison baseline. The proposed SA-HMF had a model size of 2.7 MB, an inference latency of 15.6 ± 3.2 ms, a memory usage of 118 MB, and a 10 s update interval.

Compared with the 10 s window update interval, all models achieved millisecond-level inference latency and met the requirement for real-time state updating. The proposed SA-HMF achieved the highest recognition performance while maintaining a small model size and moderate memory usage, making it suitable for deployment on a Raspberry Pi 4B-class gateway or an equivalent low-power edge device. Because state recognition and preliminary application layer inference can be performed locally without uploading raw bedroom sensing data, this deployment strategy also helps reduce privacy risks in nocturnal bedroom scenarios.

### 3.5. Exploratory Downstream Application Results

**Figure 10:**
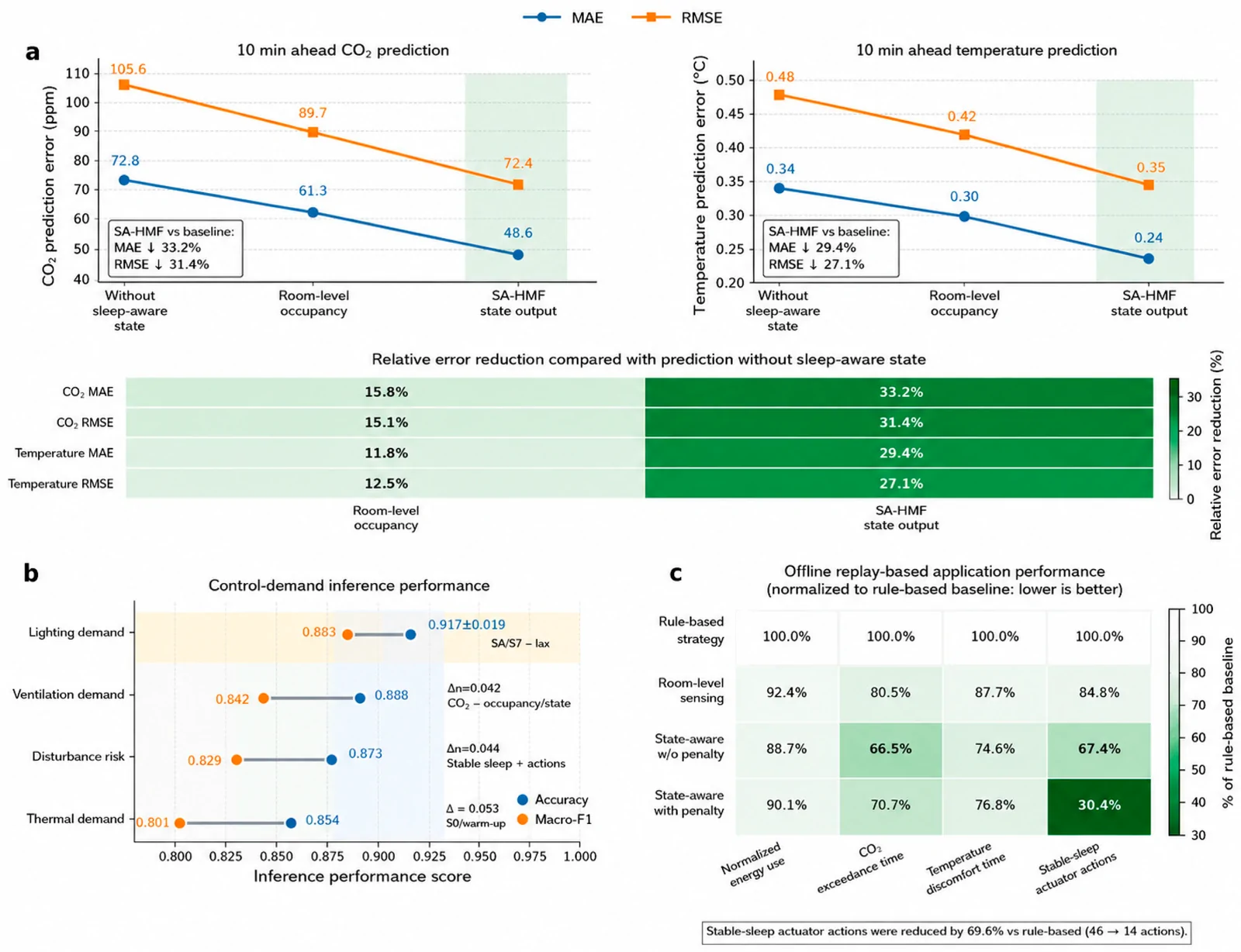
Exploratory downstream analyses based on SA-HMF outputs. (**a**) Ten-minute-ahead CO_2_ and air temperature prediction under three state information settings. (**b**) Performance on four rule-defined application proxies. (**c**) Retrospective offline replay under predefined rules; no prospective control actions were implemented.

Figure 10 summarizes the exploratory downstream analyses based on SA-HMF outputs. These analyses examined whether S0–S7 state information provided additional contextual value beyond conventional room-level occupancy and were not intended as prospective closed-loop control evaluation. As shown in Figure 10a, CO_2_ MAE decreased from 72.8 ppm without sleep-aware information to 61.3 ppm with room-level occupancy and 48.6 ppm with SA-HMF outputs. Corresponding temperature MAE values were 0.34, 0.30, and 0.24 °C, respectively, indicating that fine-grained state information provided additional context for short-term environmental prediction. For the four rule-defined proxy tasks (Figure 10b), Macro-F1 ranged from 0.801 for thermal regulation demand to 0.883 for lighting demand; ventilation demand and actuator disturbance risk achieved 0.842 and 0.829, respectively. Because these labels were rule-defined, they provide application-oriented feasibility evidence rather than direct validation of occupant demand or disturbance.

The retrospective replay in Figure 10c was based only on recorded logs and predefined rules and issued no prospective control actions. It therefore illustrates potential downstream relevance but does not establish controller effectiveness or real-world energy–IEQ–disturbance trade-offs.

### 3.6. Error Analysis and Case Studies

To better understand the sources of state recognition and event extraction errors, representative window-level errors from the held-out test set and representative event-level errors from the retrospective full-dataset analysis were examined. The complete case summary is provided in Supplementary Table S11.

The first error type was misclassification of $S_{2}$ awake-in-bed as $S_{4}$ stable sleep state, accounting for 2.8% of the $S_{2}$ windows. This usually occurred when participants were awake but remained still under low-illuminance conditions, making the bed-load and bed-motion patterns similar to those of stable sleep.

The second error type was misclassification of $S_{3}$ pre-sleep activity as $S_{7}$ wake-up transition, accounting for 4.6% of the $S_{3}$ windows. Both states may involve bed motion and illuminance changes, and their main distinction lies in temporal context within the sleep process. Strengthening elapsed-time and sleep-phase features may help reduce this confusion.

The third error type involved missed detections or boundary-matching failures for short nocturnal out-of-bed events. These events were usually brief or lacked door-contact activation, relying mainly on bed-load reduction and weak PIR activity. As a result, they could be misclassified as $S_{1}$ occupied-out-of-bed or short $S_{4}/S_{5}$ segments. Five representative cases were further inspected. A dedicated transition detector or shorter event detection window may help improve this issue.

The fourth error type was confusion between $S_{5}$ movement/turning and brief awakening or potential discomfort-related movement, accounting for 6.1% of the $S_{5}$ windows. Because subjective comfort feedback and clinical sleep labels were not collected, the model could not reliably distinguish ordinary turning, brief awakening, and discomfort-induced movement. Future work may incorporate lightweight subjective feedback, personalized thresholds, or longer-term data for adaptive modeling.

Overall, errors were concentrated in ambiguous boundary states and short transition events, rather than in stable states or clear out-of-bed events. This is consistent with the class-wise and event-level results: SA-HMF performed reliably for $S_{0}$, $S_{4}$, and in-bed/out-of-bed transitions, but there remains room for improvement in awake transition states such as $S_{2}/S_{3}/S_{7}$ and short $S_{6}$ events. These findings indicate that the proposed furniture-embedded multimodal sensing framework provided stable sleep-related state recognition in the tested nocturnal bedroom setting while also supporting event detection, edge deployment, and exploratory downstream application analysis.

## 4. Discussion

This study developed a privacy-preserving bed-centered sensing framework and SA-HMF for operational bedroom state recognition. Under the primary subject-independent split, SA-HMF achieved 0.925 Accuracy and 0.874 Macro-F1. Compared with recent unobtrusive smart bed and multimodal sleep recognition studies [36,37], the present work focuses on eight operational bedroom states rather than sleep posture or conventional sleep-state assessment. Its main contribution lies in the task-specific organization of bed-interaction, bedside IEQ, room-context, temporal cues, and sensing quality information within a unified recognition framework.

The results confirm that bed-interaction sensing provides the primary physical evidence for fine-grained bedroom state recognition. Room-level sensors can indicate occupancy and environmental changes but cannot directly distinguish in-bed, stable, movement, or bed-exit conditions. Accordingly, the bed-only model outperformed the room-level sensing model, while complete bed node dropout caused the largest performance degradation. PIR, door-contact, temporal, and IEQ information provided complementary cues, particularly for occupied-out-of-bed, nocturnal out-of-bed, and wake-up transition states.

Controlled comparisons further supported the bed-conditioned hierarchy. The full and non-hierarchical models differed by only 2.63% in parameter count, while the full model improved LOSO Macro-F1 by 0.017 (95% CI: 0.006–0.028). The improvement over the model without attention/gating was smaller (0.010; 95% CI: −0.001–0.020). Thus, the present evidence more strongly supports the asymmetric bed-conditioned hierarchy, whereas the incremental benefit of attention/gating remains modest at the current sample size.

SA-HMF also supported transition event extraction, robustness analysis, and edge deployment. In-bed transition, final out-of-bed transition, nocturnal out-of-bed event, and wake-up transition were extracted from the state probability sequences, with event-level F1-scores ranging from 0.846 to 0.950. The optimized model required 15.6 ± 3.2 ms per inference, well below the 10 s update interval. Together with the sensing degradation experiments, these results support local and privacy-preserving deployment, although practical performance remains sensitive to bed node installation, sensor placement, communication quality, synchronization, and sensor degradation.

The exploratory application layer analyses suggest that SA-HMF state outputs can provide additional contextual information for downstream smart bedroom applications. State information improved short-term environmental prediction and supported the rule-defined proxy tasks. However, these analyses were retrospective and application-oriented. In particular, the offline replay did not issue real-time control commands and should not be interpreted as evidence of prospective closed-loop control effectiveness or real-world energy–IEQ–disturbance trade-offs.

The S0–S7 scheme should also be interpreted as an operational state system rather than a universal sleep taxonomy. Its constituent concepts are observable, but their organization into eight states was designed for the present sensing configuration and application context. The results therefore support operational usefulness within the tested framework rather than universal validity of the state number or boundaries.

The generalizability of this study remains limited. All data were collected in one experimental bedroom using the same bed, sensor layout, and installation protocol. Thus, the subject-independent and LOSO evaluations demonstrate transfer to unseen participants only under the tested environment and sensing configuration. Changes in room geometry, bed properties, sensor placement, device specifications, or calibration may alter multimodal signal relationships and recognition performance. Validation across different bedrooms, beds, sensing installations, seasons, and populations is therefore required.

Data independence should be interpreted at the participant level rather than the window level. Adjacent 30 s windows generated with a 10 s stride shared 20 s of data and were therefore correlated. Accordingly, participant-level inference was used, and random window splitting was retained only as a potentially optimistic reference. With non-overlapping 30 s windows, Macro-F1 decreased only from 0.874 to 0.865, while the advantage over the non-hierarchical model was preserved. Thus, window overlap increased sampling density but did not solely explain the main performance pattern.

Other limitations include the limited number of nocturnal out-of-bed events, the absence of clinical sleep labels and subjective comfort feedback, and the exploratory nature of the downstream application analysis. Future work should prioritize multi-bedroom and multi-population validation, longer-term collection of low-frequency transition events, and cross-room adaptation and personalization.

## 5. Conclusions

This study developed a privacy-preserving bed-centered sensing framework and a bed-conditioned, quality-aware asymmetric SA-HMF architecture for eight operational bedroom states. Under the primary subject-independent split, SA-HMF achieved 0.925 Accuracy and 0.874 Macro-F1. Participant-level LOSO analysis supported the bed-centered hierarchy, whereas the additional attention/gating improvement was positive but not statistically conclusive. A non-overlapping window analysis retained a Macro-F1 of 0.865 and preserved the structural ordering.

The framework also supported event extraction, sensing degradation evaluation, edge inference, and exploratory downstream application analysis. These findings demonstrate feasibility and transfer to unseen healthy participants within the tested bedroom and sensing configuration. They do not establish universal validity of S0–S7 or external transportability across bedrooms and populations.

## Figures and Tables

**Table 1:** Classification of sensing and recognition strategies for sleep-related smart bedroom monitoring.

Strategy	Typical Inputs	Advantages	Limitations/Role in This Study
Room-level ambient sensing	PIR, door contact, CO_2_, temperature, humidity, illuminance [6,7,8]	Low-cost, easy to deploy, privacy-preserving	Cannot observe bed interactions; used as IEQ/context cues
Wearable sensing	Heart rate, movement, skin temperature, sleep/wake cues [9,10]	Provides physiological information	May affect comfort and adherence; not used as core input
Bed-based sensing	Pressure, load, piezoelectric, bed-motion, accelerometry [11,12,18,20,21]	Captures in-bed status, bed-exit, stability, and movement	Limited room-context information; used as the bed-interaction branch
Contactless sensing	Radar, Wi-Fi, infrared array, depth/vision sensing [13,14,15]	Enables non-contact presence or motion sensing	May involve privacy, occlusion, or deployment issues
Multimodal sleep monitoring	Pressure, accelerometry, environmental, thermal, or physiological signals [17,18,19,20]	Combines complementary information	Often targets sleep stages, posture, or respiration, not control states
Smart home activity recognition	Ambient sensor networks, routine/workflow models [22]	Supports activity and routine inference	Usually focuses on daytime or general activities
Proposed framework	Bed load, bed accelerometry, bedside IEQ, PIR, door contact, time features	Combines bed interaction, environment, context, and time	Supports S0–S7 control-oriented bedroom state recognition

**Table 2:** Overview of the experimental bedroom dataset.

Item	Description
Participants	20 healthy adults; 10 males/10 females
Age range	19–53 years; mean age 31.8 ± 8.6 years
Planned recordings	140 participant-nights
Successfully recorded nights	137 participant-nights
Valid nights after night-level and long-segment quality control	127 participant-nights
Total raw recording duration	1094.8 h, including invalid nights or segments later excluded
Recording range per night	From bedroom entry or pre-sleep activity to a short period after final out-of-bed and room-exit events the next morning
Window length/sliding stride	30 s/10 s
Candidate windows before window-level quality screening	365,921 windows, generated from synchronized segments retained after night-level and long-segment quality control
Candidate window stride-based equivalent coverage	1016.4 h, calculated as 365,921 × 10 s/3600; used only to indicate overlapping sample scale
Windows excluded by window-level screening	23,237 windows, accounting for approximately 6.35% of candidate windows
Excluded window stride-based equivalent coverage	64.5 h, calculated as 23,237 × 10 s/3600
Final valid labeled windows	342,684 windows for model training, validation, and testing; approximately 93.65% of candidate windows
Valid window stride-based equivalent duration	951.9 h, calculated as 342,684 × 10 s/3600; used only to indicate final overlapping sample scale
State labels	Eight control-oriented sleep-related bedroom states, S0–S7
Nocturnal out-of-bed events	41 events
Wake-up transition events	112 events
Valid nights with environmental variations	43 nights
Edge deployment log coverage	127 valid nights
Data-splitting protocols	Subject-independent split, night-independent split, random window split, and leave-one-subject-out cross-validation

**Table 3:** Experimental scenarios and purposes.

Scenario	Purpose	Data Source/Implementation
Natural sleep nights	To collect stable sleep-related states, movement, and state transitions during natural sleep	Participants followed their usual sleep and wake-up routines
Pre-sleep activity	To distinguish awake-in-bed and pre-sleep activity states	Natural behaviors such as reading, phone use, bed preparation, and being awake after getting into bed were recorded
Nocturnal out-of-bed event	To detect nocturnal leaving-bed and returning-to-bed events	Mainly based on natural nocturnal events, confirmed by bed-load reduction, PIR activity, and door-contact events
Wake-up transition	To identify gradual morning wake-up states	Bed-motion, illuminance, and bed-interaction changes from awakening to final out-of-bed event were recorded
Environmental variation	To support analysis of CO_2_, temperature, and illuminance changes in state recognition and downstream prediction	Environmental changes caused by natural ventilation, air-conditioning, lighting changes, and room entry/exit were recorded
Robustness analysis under sensor degradation	To evaluate robustness to missing data, node failure, noise, and drift	Random missingness and modality dropout were generated by offline masking; sensor noise and drift were evaluated using noisy or drift-affected segments observed in the recordings

**Table 4:** Control-oriented S0–S7 sleep-related bedroom state system.

State ID	English Label	State Definition	Main Labeling Evidence	Control Implication
S0	Unoccupied	No occupant in the bedroom	Low bed load, no PIR activity, and door-contact or room-exit events supporting an unoccupied state	Energy-saving mode
S1	Occupied-out-of-bed	Occupant present in the bedroom but not in bed	Low bed load, PIR activity, and door-contact or room activity events supporting room occupancy	Basic comfort maintenance
S2	Awake-in-bed	Occupant awake in bed	Bed-load signal indicates in-bed status, with low or moderate bed motion, occurring during non-transition pre-sleep or post-wake periods	Pre-sleep comfort mode
S3	Pre-sleep activity	Pre-sleep activity	Activity in bed or near the bed, relatively high illuminance, evident bed motion or room activity, and occurrence before sleep onset	Lighting and thermal comfort support
S4	Stable sleep-related state	Stable sleep-related state	Stable bed-load pattern, low accelerometry-derived bed motion, and occurrence during the main nocturnal sleep period	Low-disturbance control
S5	Movement/turning	Body movement or turning	Marked bed-load or bed-motion fluctuations while the occupant remains in bed, typically with a short duration	Potential discomfort check
S6	Nocturnal out-of-bed	Nocturnal out-of-bed episode	Transition from in-bed to out-of-bed event during the night, bed-load reduction, PIR or door-contact events, followed by return to bed	Dim night-light or safety assistance
S7	Wake-up transition	Wake-up transition	Increased bed-motion and illuminance changes in the morning, followed by final out-of-bed or room-exit state	Environmental preconditioning and gradual lighting

**Table 5:** Rule-defined application layer proxy tasks used for exploratory evaluation.

Demand Type	Inputs	Output
Ventilation demand	CO_2_, occupancy probability, sleep-related states	low/medium/high
Thermal regulation demand	Air temperature, relative humidity, sleep-related states	no action/cooling adjustment/heating adjustment
Lighting demand	Illuminance, nocturnal out-of-bed state, wake-up transition state	off/night light/gradual light
Actuator disturbance risk	Sleep-related states, actuator action changes	low/high

**Table 6:** Baseline methods and controlled ablation settings.

Type	Setting	Input or Structural Configuration	Evaluation Purpose
Baseline	Rule-based method	Bed-load thresholds, PIR and door-contact events, and time-based rules	Represents conventional threshold and event logic recognition
Baseline/modality ablation	Bed-only model	Bed-load and accelerometry-derived bed-interaction features only	Evaluates the standalone contribution of furniture-embedded bed-interaction sensing
Baseline	Room-level sensing model	IEQ, PIR, door-contact, and time features; no bed-interaction input	Evaluates the limitations of conventional room-level sensing
Baseline	Early-fusion LightGBM	Direct concatenation of all core window-level features; no intermediate bed-interaction representation	Represents conventional feature-level multimodal fusion
Baseline	GRU temporal model	Temporal processing of concatenated multimodal feature sequences; no explicit bed-conditioned hierarchy	Represents a temporal deep learning baseline
Modality ablation	IEQ-only	CO_2_, air temperature, relative humidity, and illuminance features only	Evaluates the standalone recognition capability of bedside environmental sensing
Modality ablation	Context + time-only	PIR, door-contact, room event, and causal time features only	Evaluates the standalone contribution of room-context and temporal information
Modality ablation	Bed + IEQ	Bed-interaction and IEQ features	Evaluates the complementarity between bed-interaction and environmental information
Modality ablation	Bed + context + time	Bed-interaction, room-context, and causal time features	Evaluates the complementarity between bed-interaction and behavioral context
Structural ablation	SA-HMF without hierarchy	Retains the modality encoders, attention/gating mechanism, hidden dimensions, classifier depth, loss, and training settings but removes the intermediate bed-interaction hierarchy and treats bed features as a parallel modality	Isolates the contribution of the bed-conditioned asymmetric hierarchy
Structural ablation	SA-HMF without attention/gate	Retains the intermediate bed-interaction hierarchy but replaces adaptive attention and quality-aware gating with direct multimodal fusion	Isolates the contribution of adaptive relevance weighting and sensing quality regulation
Full model	Full SA-HMF	Intermediate bed-interaction representation, bed-conditioned multimodal weighting, and quality-aware gating	Complete proposed architecture

**Table 7:** Overall state recognition performance under the subject-independent split.

Method	Accuracy	Macro-F1	Weighted-F1	Balanced Accuracy
Rule-based method	0.781	0.558	0.766	0.604
Bed-only model	0.844	0.694	0.839	0.731
Room-level sensing model	0.773	0.571	0.761	0.609
Early-fusion LightGBM	0.887	0.807	0.888	0.834
GRU temporal model	0.904	0.833	0.906	0.853
SA-HMF without hierarchy	0.913	0.856	0.915	0.856
Proposed SA-HMF	0.925	0.874	0.927	0.864

**Table 8:** Retrospective event extraction performance across all 127 valid participant-nights.

Event	Number of Events	Precision	Recall	F1-Score	Mean Detection Delay
In-bed transition	127	0.957	0.944	0.950	5.8 ± 3.1 s
Final out-of-bed transition	127	0.941	0.929	0.935	7.2 ± 4.0 s
Nocturnal out-of-bed event	41	0.864	0.829	0.846	11.6 ± 7.4 s
Wake-up transition	112	0.873	0.848	0.860	26.4 ± 18.2 s

**Table 9:** Modality contribution and structural ablation results.

Model Variant	Bed	IEQ	Context	Time	Hierarchy	Attention/Gate	Macro-F1
Bed-only	Yes	—	—	—	—	—	0.694
IEQ-only	—	Yes	—	—	—	—	0.438
Context-only	—	—	Yes	Yes	—	—	0.582
Bed + IEQ	Yes	Yes	—	—	—	—	0.753
Bed + context + time	Yes	—	Yes	Yes	—	—	0.826
Bed + IEQ + context + time	Yes	Yes	Yes	Yes	—	—	0.846
SA-HMF without hierarchy	Yes	Yes	Yes	Yes	—	Yes	0.856
SA-HMF without attention/gate	Yes	Yes	Yes	Yes	Yes	—	0.861
Proposed SA-HMF full model	Yes	Yes	Yes	Yes	Yes	Yes	0.874

**Table 10:** Summary comparison and interpretation of validation protocols.

Validation Protocol	Accuracy	Macro-F1	Weighted-F1	Interpretation
Random window split	0.949	0.911	0.951	Reference only; potentially optimistic because overlapping neighboring windows may occur in both training and test sets
Night-independent split	0.931	0.884	0.932	Cross-night stability within the same participants, bedroom, and sensing configuration
Subject-independent split	0.925	0.874	0.927	Primary protocol; transfer to unseen participants within the tested bedroom and sensing configuration
LOSO CV	0.914 ± 0.029	0.852 ± 0.043	0.916 ± 0.030	Participant-level variability and uncertainty within the tested setting; not cross-bedroom validation

**Table 11:** Edge deployment performance. Note: The unoptimized SA-HMF implementation refers to an engineering implementation without the final edge-optimized inference configuration. It was used only for deployment overhead comparison and was not treated as an additional state recognition algorithm baseline.

Model	Model Size	Inference Latency	Memory Usage	Update Interval	Suitable for Edge Deployment
LightGBM	4.8 MB	6.4 ± 1.2 ms	74 MB	10 s	Yes
GRU temporal model	1.9 MB	18.7 ± 3.6 ms	132 MB	10 s	Yes
Unoptimized SA-HMF implementation	3.4 MB	21.8 ± 4.1 ms	146 MB	10 s	Yes
Proposed SA-HMF	2.7 MB	15.6 ± 3.2 ms	118 MB	10 s	Yes

## Data Availability

The raw data generated and analyzed during this study are not publicly available because they contain privacy-sensitive nocturnal bedroom sensing records from human participants and are subject to participant privacy protection and consent-scope limitations. To protect participant privacy, raw bedroom sensing streams, event logs, and data that may reveal individual nocturnal routines or bedroom behavior patterns cannot be shared publicly. De-identified window-level feature data, label files, model configuration files, and analysis scripts supporting the findings of this study are available from the corresponding author upon reasonable request, subject to institutional approval, participant-consent restrictions, and a data-use agreement. The supplementary tables supporting this article are provided as Supplementary Materials.

## Supplementary Materials

The following supporting information can be downloaded at https://www.mdpi.com/article/10.3390/s26185743/s1: Table S1: Core sensing inputs for S0–S7 state recognition; Table S2: Separation between S0–S7 state-recognition inputs and application layer variables; Table S3: Hierarchical recognition structure of SA-HMF; Table S4: Sleep-aware state descriptors derived from SA-HMF; Table S5: Data-splitting protocols and purposes; Table S6: Metrics for state recognition, event detection and generalization evaluation; Table S7: Metrics for robustness, deployment and application layer evaluation; Table S8: Dataset characteristics and signal quality summary; Table S9: Data quality of sensing modalities; Table S10: Class-wise recognition performance of proposed SA-HMF; Table S11: Error analysis and representative cases. Table S12: Event extraction threshold sensitivity analysis for nocturnal out-of-bed and wake-up transition detection. Table S13: Controlled structural configurations and state-specific results. Table S14: Participant-level paired LOSO comparison of structural variants. Table S15: Sensitivity to window overlap.
