# Supplementary material for: Smart Furniture-Embedded Bed-Interaction and Multimodal Room Sensing with Sleep-Aware Hierarchical Fusion for Control-Oriented Bedroom State Recognition
Source: Sensors (Basel). 2026 Sep 10;26(18):5743. doi: 10.3390/s26185743 (PMC13611615; doi:10.3390/s26185743)
Supplement: Supplementary file 1 [file sensors-26-05743-s001.zip › sensors-4379424-supplementary.pdf]

Supplementary Materials

Supplementary Table S1. Core sensing inputs for S0–S7 state recognition.

Module	Retained input	Data field	Sampling / recording mode	Used in S0–S7 main model	Role in state recognition	Role in application layer
Bed-interaction node	Bed pressure / load	P_bed or P_total	5–10 Hz	Yes	Identify in-bed, out-of-bed, stable sleep and nocturnal out-of-bed states	Derive in-bed probability and stable-sleep context
Bed-interaction node	Bed vibration / acceleration	A_bed or Acc_rms	10–25 Hz	Yes	Identify movement, turning, sleep instability and pre-wake activity increase	Support sleep-sensitive state and disturbance-risk inference
Bedside IEQ node	CO_2 concentration	CO_2	Every 10 s	Yes	Provide auxiliary occupancy and state-transition cues	Support ventilation-related prediction and ventilation-demand inference
Bedside IEQ node	Air temperature	T_air	Every 10 s	Yes	Provide thermal-environment context	Support temperature prediction and thermal-demand inference
Bedside IEQ node	Relative humidity	RH	Every 10 s	Yes	Describe hygrothermal conditions	Support thermal comfort and thermal-demand inference
Bedside IEQ node	Illuminance	Lux	Every 10 s	Yes	Support recognition of pre-sleep activity, nocturnal out-of-bed and wake-up transition	Support lighting-demand inference
Room-context node	PIR motion	PIR	Event-based / converted every 10 s	Yes	Distinguish unoccupied, occupied-out-of-bed and room activity	Support occupancy probability and control-demand inference
Room-context node	Door contact	Door	Event-based / converted every 10 s	Yes	Identify room entry/exit, nocturnal leaving and return-to-room events	Support nocturnal out-of-bed and return-related application inference
Time features	Clock time, elapsed time after going to bed or lights off, nighttime/morning phase	Time, T_elapsed, Phase_time	Generated for each window	Yes	Support distinction among pre-sleep, nighttime, nocturnal out-of-bed and wake-up transition states	Provide temporal context for environmental prediction and application-layer demand inference

Supplementary Table S2. Separation between S0–S7 state-recognition inputs and application-layer variables.

Symbol	Meaning	Used in S0–S7 main state-recognition model	Purpose
X_{bed,t}	Bed pressure/load, bed vibration/acceleration, in-bed status, movement intensity and pressure stability features	Yes	Recognize in-bed, out-of-bed, stable sleep, movement/turning and nocturnal out-of-bed states
X_{IEQ,t}	CO_2, air temperature, relative humidity, illuminance and their window-level means, rates of change and trend features	Yes	Provide environmental context for state recognition and support downstream environmental prediction
X_{ctx,t}	PIR status, door-contact events, room-activity events and room-entry/exit events	Yes	Support distinction among unoccupied, occupied-out-of-bed, nocturnal leaving and return-to-room states
X_{time,t}	Clock time, elapsed time after going to bed or lights off, time since the latest past bed/context event and nighttime/morning phase indicators; future event timing such as final wake-up or final out-of-bed is excluded	Yes	Support distinction among pre-sleep, nighttime, nocturnal out-of-bed and wake-up transition states while avoiding future-information leakage
p̂_t	S0–S7 state-probability vector or sleep-aware state descriptors output by SA-HMF	No; model output	Provide state context for application-layer environmental prediction and control-demand inference
X_{act,t}	Lighting, air-conditioning and ventilation states and actuator-action changes	No	Used for control-demand inference and disturbance-risk analysis
X_{energy,t}	Measured device power, cumulative energy and normalized measured-energy variables	No	Used for measured-energy-based offline replay and energy-related application evaluation

Supplementary Table S3. Hierarchical recognition structure of SA-HMF.

Module	Input	Output	Function	Relationship to S0–S7 state recognition
Bed-interaction representation layer	Bed pressure/load and bed vibration/acceleration	Bed-interaction representations related to in-bed, out-of-bed, movement/turning and stability states	Capture direct human–bed interaction	Part of the SA-HMF main classifier
Bedroom-state classification layer	Bed-interaction representation, bedside IEQ, PIR, door contact and time features	S0–S7 sleep-related bedroom state probabilities	Recognize control-oriented sleep-related bedroom states	Part of the SA-HMF main classifier and the final output of the main model
Downstream application layer	S0–S7 state probabilities and derived state descriptors, IEQ variables, actuator logs and energy records	CO_2 and temperature prediction results, ventilation demand, thermal demand, lighting demand and disturbance risk	Support environmental prediction, control-demand inference and offline replay application evaluation	Not part of the S0–S7 main classifier; actuator and energy variables are not used to train the main state-recognition model

Supplementary Table S4. Sleep-aware state descriptors derived from SA-HMF.

Output	Meaning	Main source	Relationship to SA-HMF main classifier
$\hat{p}_t$	S0–S7 state-probability vector	Bed-interaction, IEQ, context and time features	Direct output of the main classifier
P_t^{occ}	Room-occupancy-related probability	S0–S7 state probabilities, PIR and door contact	Derived from state probabilities and context information
P_t^{bed}	In-bed probability	S2, S3, S4, S5 and S7 probabilities, bed pressure/load	Derived from state probabilities and bed-interaction information
P_t^{sleep}	Stable-sleep probability	S4 stable-sleep probability and bed stability	Derived from state probabilities
P_t^{move}	Movement/turning probability	S5 movement/turning probability and bed vibration/acceleration	Derived from state probabilities
P_t^{trans}	Nocturnal out-of-bed or wake-up transition probability	S6 nocturnal out-of-bed and S7 wake-up transition probabilities	Derived from state probabilities

Supplementary Table S5. Data-splitting protocols and purposes.

Protocol	Setting	Purpose
Random window split	All valid windows were randomly split into 70% / 15% / 15%	Provide a basic performance reference, but not used as the main conclusion
Night-independent split	Different nights from the same participant were assigned to training, validation and test sets	Evaluate cross-night generalization
Subject-independent split	14 participants for training, 3 for validation and 3 for testing	Evaluate cross-subject generalization and serve as the primary result
Leave-one-subject-out cross-validation	One participant was left out as the test subject in each fold	Evaluate stability under inter-individual differences

Supplementary Table S6. Metrics for state recognition, event detection and generalization evaluation.

Evaluation target	Metric	Description
Window-level overall recognition	Accuracy, macro-F1, weighted-F1 and balanced accuracy	Evaluate overall S0–S7 recognition performance
Class-wise recognition	Precision, Recall and F1-score for S0–S7	Analyze state-specific recognition performance
Low-frequency key states	S6 F1, S7 F1, macro-F1 and balanced accuracy	Reduce majority-class dominance in performance interpretation
Confusion analysis	Confusion matrix	Analyze major confused state combinations
Event detection	Event-level Precision, Recall and F1-score	Evaluate in-bed, final out-of-bed, nocturnal out-of-bed and wake-up transition detection
Temporal boundary estimation	Detection delay and onset error	Evaluate event-triggering time and boundary estimation
Generalization ability	Subject-independent split, night-independent split, random window split and LOSO CV	Evaluate stability across subjects, nights and inter-individual differences

Supplementary Table S7. Metrics for robustness, deployment and application-layer evaluation.

Evaluation target	Metric	Description
Sensing-network quality	Missing rate, packet loss and synchronization error	Evaluate the reliability of multi-node data acquisition
Missing-data robustness	Accuracy and macro-F1 under random missingness and modality dropout	Evaluate model adaptability to missing data and node failure
Key-class robustness	S6 F1 and S4 F1	Evaluate the stability of low-frequency events and stable-sleep state under perturbations
Edge inference	Model size, inference latency and memory usage	Evaluate local-gateway deployability
Real-time capability	Update interval and inference delay relative to the 10 s stride	Determine whether real-time state updating is supported
CO_2 prediction	MAE and RMSE	Evaluate short-term CO_2 prediction error
Temperature prediction	MAE and RMSE	Evaluate short-term air-temperature prediction error
Control-demand inference	Accuracy and macro-F1	Evaluate ventilation, thermal, lighting and disturbance-risk demand inference
Measured-energy-based offline replay	Normalized measured energy use, CO_2 exceedance time and temperature discomfort time	Evaluate application-layer performance based on measured power or cumulative energy
Disturbance-aware behavior	Actuator actions during stable sleep	Evaluate whether device actions during stable sleep are reduced

Supplementary Table S8. Dataset characteristics and signal-quality summary.

Note: Candidate windows and valid labeled windows were generated using a 30 s window length and a 10 s stride. The stride-based equivalent coverage/duration was calculated as number of windows × 10 s / 3600 and is used only to describe the scale of overlapping window samples; it should not be interpreted as non-overlapping real recording duration.

Item	Value	Description
Number of participants	20	Healthy adults, 10 males and 10 females
Age range	19–53 years	Mean ± SD: 31.8 ± 8.6 years
Scheduled participant-nights	140	20 × 7
Successfully recorded nights	137	Three nights were not completely recorded due to device or participant-related issues
Valid participant-nights after night-/segment-level QC	127	Equivalent to 92.7% of successfully recorded nights
Total raw recording duration	1094.8 h	Includes some nights or segments that were later excluded
Window length / stride	30 s / 10 s	Window setting for state recognition
Candidate windows before window-level filtering	365,921	Generated by windowing synchronized segments retained after night-/segment-level quality control
Candidate-window stride-based equivalent coverage	1016.4 h	Describes only the stride-based equivalent scale of candidate windows
Window-level removed windows	23,237	Windows removed because of missing data, anomalies or uncertain labels; approximately 6.35% of candidate windows
Removed-window stride-based equivalent coverage	64.5 h	Describes only the stride-based equivalent scale of removed windows
Valid labeled windows after window-level QC	342,684	Final modeling windows after window-level quality filtering and label checking; approximately 93.65% of candidate windows
Valid-window stride-based equivalent duration	951.9 h	Describes only the stride-based equivalent scale of final valid labeled windows
Label classes	S0–S7	Eight control-oriented sleep-related bedroom states
Nocturnal out-of-bed events	41	Low-frequency key events
Wake-up transition events	112	Wake-up transitions were incomplete in some nights
Environmental variation nights	43	Nights with noticeable CO_2, temperature or illuminance variation
Edge-deployment log nights	127	Covering all valid nights

Supplementary Table S9. Data quality of sensing modalities.

Modality	Data coverage	Missing rate	Notes
Bed pressure / load	97.8%	2.2%	Small number of missing samples caused by bed-node reboot
Bed acceleration / vibration	96.9%	3.1%	Short wireless packet loss occurred in a few nights
CO_2	95.6%	4.4%	Missing samples mainly caused by CO_2 module warm-up and communication delay
Air temperature	98.1%	1.9%	Generally stable
Relative humidity	98.0%	2.0%	Measured by the same IEQ node as air temperature
Illuminance	97.3%	2.7%	A few segments were affected by temporary shading
PIR	98.6%	1.4%	Stable event-based sensor
Door contact	99.1%	0.9%	Most stable sensing modality
Mean packet loss rate	1.84%	—	Gateway-level statistic
Mean synchronization error	0.46 ± 0.31 s	—	Below the 2 s threshold

Supplementary Table S10. Class-wise recognition performance of Proposed SA-HMF.

State	Precision	Recall	F1-score	Main explanation
S0 Unoccupied	0.951	0.941	0.946	Unoccupied state was relatively easy to recognize
S1 Occupied-out-of-bed	0.858	0.825	0.841	Prone to confusion with S6
S2 Awake-in-bed	0.874	0.851	0.862	Prone to confusion with S3 / S7
S3 Pre-sleep activity	0.846	0.824	0.835	Pre-sleep boundaries were relatively ambiguous
S4 Stable sleep	0.962	0.976	0.969	Large sample size and stable bed-interaction signals
S5 Movement / turning	0.876	0.846	0.861	Overlapped with short awake episodes
S6 Nocturnal out-of-bed	0.835	0.790	0.812	Low-frequency event with slightly lower recall
S7 Wake-up transition	0.880	0.857	0.868	Confused with S2 awake-in-bed
Macro average	0.885	0.864	0.874	—

Supplementary Table S11. Error analysis and representative cases.

Error case	Frequency in test set	Main confused classes	Possible reason	Potential improvement
Awake-in-bed misclassified as stable sleep	2.8% of S2 windows	S2 → S4	Low movement and low illuminance made bed-interaction signals resemble stable sleep	Add phone-interaction or respiratory micro-motion features
Pre-sleep activity confused with wake-up transition	4.6% of S3 windows	S3 → S7	Both states may show illuminance changes and bed movement	Strengthen elapsed-time-after-bedtime features
Short nocturnal out-of-bed missed	Five representative cases examined	S6 → S1 / S4	Short event duration and no door-contact trigger	Add a dedicated transition detector
Movement/turning confused with short awake/discomfort-like episodes	6.1% of S5 windows	S5 → S4 / S2	Subjective comfort feedback was unavailable	Add comfort feedback or adaptive labels

Supplementary Table S12. Event-extraction threshold sensitivity analysis for nocturnal out-of-bed and wake-up transition detection.

Analysis setting	θ6	θ7	K	Nocturnal out-of-bed F1	Wake-up transition F1	Mean delay for nocturnal out-of-bed	Mean delay for wake-up transition
Lower θ6	0.30	0.40	3	0.813	—	8.9 ± 6.8 s	—
Lower θ6	0.35	0.40	3	0.832	—	10.2 ± 7.1 s	—
Main setting	0.40	0.40	3	0.846	0.860	11.6 ± 7.4 s	26.4 ± 18.2 s
Higher θ6	0.45	0.40	3	0.837	—	13.8 ± 8.5 s	—
Higher θ6	0.50	0.40	3	0.814	—	16.7 ± 10.1 s	—
Lower θ7	0.40	0.30	3	—	0.831	—	21.8 ± 16.4 s
Lower θ7	0.40	0.35	3	—	0.849	—	24.1 ± 17.3 s
Higher θ7	0.40	0.45	3	—	0.852	—	29.2 ± 19.5 s
Higher θ7	0.40	0.50	3	—	0.829	—	34.7 ± 22.6 s
No smoothing	0.40	0.40	1	0.821	0.833	8.1 ± 6.2 s	20.7 ± 15.8 s
Longer smoothing	0.40	0.40	5	0.839	0.851	16.3 ± 9.6 s	33.1 ± 20.4 s

Supplementary Table S13. Controlled structural configurations and state-specific results.

Model	Parameters	Macro-F1	S1 F1	S6 F1	S7 F1	IEQ-node dropout Macro-F1	Context-node dropout Macro-F1
SA-HMF without hierarchy	654,730	0.856	0.834	0.781	0.839	0.836	0.810
SA-HMF without attention/gate	660,982	0.861	0.846	0.793	0.851	0.839	0.817
Full SA-HMF	672,418	0.874	0.862	0.812	0.868	0.857	0.843

Supplementary Table S14. Participant-level paired LOSO comparison of structural variants.

Model	Macro-F1 mean ± SD	Median	95% CI	Difference from full	Paired 95% CI	Full better	Holm-adjusted p
Without hierarchy	0.835 ± 0.047	0.841	0.814–0.855	0.017	0.006–0.028	16/20	0.024
Without attention/gate	0.842 ± 0.045	0.848	0.822–0.861	0.010	−0.001–0.020	14/20	0.082
Full SA-HMF	0.852 ± 0.043	0.858	0.833–0.870	Reference	—	—	—

Supplementary Table S15. Sensitivity to window overlap.

Window setting	Model	Valid windows	Accuracy	Macro-F1	Balanced Accuracy
30 s / 10 s stride	Without hierarchy	342,684	0.913	0.856	0.856
30 s / 10 s stride	Full SA-HMF	342,684	0.925	0.874	0.864
30 s / 30 s stride	Without hierarchy	114,126	0.906	0.848	0.846
30 s / 30 s stride	Full SA-HMF	114,126	0.918	0.865	0.856
